# On the Rollout of Network Slicing in Carrier Networks: A Technology Radar

**DOI:** 10.3390/s21238094

**Published:** 2021-12-03

**Authors:** Jose Ordonez-Lucena, Pablo Ameigeiras, Luis M. Contreras, Jesús Folgueira, Diego R. López

**Affiliations:** 1 CTIO Unit, Telefónica I+D, 28050 Madrid, Spain; luismiguel.contrerasmurillo@telefonica.com (L.M.C.); jesus.folgueira@telefonica.com (J.F.); diego.r.lopez@telefonica.com (D.R.L.); 2 Department of Signal Theory, Telematics and Communications, University of Granada, 18071 Granada, Spain; pameigeiras@ugr.es

**Keywords:** end-to-end network slicing, 5G, smart networks and services, multi-domain orchestration, technology radar, roll-out plan

## Abstract

Network slicing is a powerful paradigm for network operators to support use cases with widely diverse requirements atop a common infrastructure. As 5G standards are completed, and commercial solutions mature, operators need to start thinking about how to integrate network slicing capabilities in their assets, so that customer-facing solutions can be made available in their portfolio. This integration is, however, not an easy task, due to the heterogeneity of assets that typically exist in carrier networks. In this regard, 5G commercial networks may consist of a number of domains, each with a different technological pace, and built out of products from multiple vendors, including legacy network devices and functions. These multi-technology, multi-vendor and brownfield features constitute a challenge for the operator, which is required to deploy and operate slices across all these domains in order to satisfy the end-to-end nature of the services hosted by these slices. In this context, the only realistic option for operators is to introduce slicing capabilities progressively, following a phased approach in their roll-out. The purpose of this paper is to precisely help designing this kind of plan, by means of a technology radar. The radar identifies a set of solutions enabling network slicing on the individual domains, and classifies these solutions into four rings, each corresponding to a different timeline: (i) as-is ring, covering today’s slicing solutions; (ii) deploy ring, corresponding to solutions available in the short term; (iii) test ring, considering medium-term solutions; and (iv) explore ring, with solutions expected in the long run. This classification is done based on the technical availability of the solutions, together with the foreseen market demands. The value of this radar lies in its ability to provide a complete view of the slicing landscape with one single snapshot, by linking solutions to information that operators may use for decision making in their individual go-to-market strategies.

## 1. Introduction

Over recent years, the telco industry has actively focused on the exploration of technologies to accelerate the roll-out of fifth generation (5G) systems worldwide. Unlike 4G, mainly focused on providing mobile broadband services to end users, 5G has been designed from its inception to help boost the digital transformation of vertical industries (i.e., industry sectors aiming at becoming fully digital, such as manufacturing, smart cities, transportation or agriculture [1]). The direct involvement of the so-called verticals and their specific needs within the 5G technology ecosystem implies the emergence of a new wave of use cases, with very different requirements in terms of performance (e.g., throughput, latency and reliability) and functionality (e.g., mobility, security, service continuity support), some of them very stringent.

To satisfy these different (and potentially conflicting) requirements in a cost-effective manner, operators need to turn their networks into programmable multi-service platforms, embracing the infrastructure and functional sharing mechanisms commonly referred to as network slicing. With network slicing, the operator’s network can be logically split into a set of programmable network partitions (i.e., network slices), each designed to satisfy a particular set of service requirements. The service-tailored logical networks resulting from this partitioning can be executed in parallel, but need to be operated in isolation from each other. This means that despite running on a common (shared) network infrastructure, network slices require separate (independent) management [2].

The technology foundations for slicing are already here. On the one hand, network functions virtualization (NFV) allows deploying the functions of every slice with necessary capacity where and when required. On the other hand, software defined networking (SDN) allows operators to programmatically steer traffic within the slice, across the deployed functions. In addition to these dynamic control means, NFV and SDN technologies also provide the ability to resize and move workloads at operation time, in such a way that the service requirements can be always met, regardless of network conditions, e.g., a faulty node or traffic load surges. However, to fully exploit the benefits that network slicing brings, it is important for the operator to apply the dynamic allocation and tailored partitioning of resources at all segments, from the radio access to the data network, including all the network domains in between. This means that the slice concept shall span the entire operator’s managed network infrastructure, resulting in the provisioning and operation of end-to-end (E2E) network slices.

The E2E nature of slicing forces operators to keep consistency in the behaviour of individual slices along the different domains. This may bring significant operational challenges in commercial networks, as outlined below:

- **Slicing readiness varies across the different domains**. In fact, the degree of penetration of slicing features in the different technology domains is not the same. For example, while the core network has incorporated network slicing support since the first 5G release (3GPP Release 15), the transport network does not support any native slicing feature yet, and first solutions have only recently been integrated into the radio access network. The main reason why the maturity level varies across technology domains (and their corresponding management domains) is mainly due to the existing fragmentation in the standardization arena, with a high number of participating Standard Development Organizations (SDOs). In the current landscape, each SDO addresses a portion of the E2E problem, developing slicing specifications for this portion under assumptions that do not necessarily match the assumptions made by other standard bodies, which typically address other portions. A clear example of this mismatching can be observed on the priorities that different SDOs set in relation to which slicing features need to be worked out in each release. In fact, these priorities are quite different across SDOs, both in time and scope.
- **Scalability burdens**. The higher the number of slices running in parallel, the heavier the burden on the operator’s OSS (Operations Support System) in terms of scalability. In fact, having a high number of instantiated tiny network slices, each requiring separate control and management, may well imply a strong impact on OSS functions (orchestration, assurance, etc.). This requires the operator to find the right balance in the slice design and activation patterns, looking to minimize this impact while properly addressing service demands. The introduction of advanced configuration and automation capabilities in OSS assets is also a must, in order to reduce the number of touches, especially in the assurance phase.
- **Multi-provider solutions**. Upcoming 5G commercial networks are to be built out of solutions from multiple technology providers. The reason for this approach is essentially related to the dangerous effect of monoculture. Single-vendor dependency is a killer for innovation, as it restricts open collaboration from the broader 5G ecosystem of companies developing new technology, use cases, and services that the market expects. In this multi-vendor ecosystem, the challenge for operators will be in the appropriate combination of pieces from different providers and in ensuring they work together, within and across domains. The high integration efforts on the operator side to achieve multi-provider interoperability can be partially relieved by selecting solutions which are standards-compliant, i.e., based on the use of open interfaces.
- **Brownfield environments**. Carrier networks are formed of already available equipment and functions (legacy is the common term for them), aimed at offering services from previous generations and even former releases of the current one. The need to keep this legacy up and running shall be combined with the introduction of the slicing functionality, avoiding the creation of silos. Unlike greenfield environments (e.g., private 5G networks), where network slicing can be easily launched as soon as commercial products are available, in carrier networks the operator needs to carefully upgrade its assets in such a way that the the legacy and slicing features can coexist. This process needs to be conducted in a cost-efficient way, ensuring that the upfront CAPEX behind every required upgrade will be compensated with a large mass of customers willing to consume the added slicing features.

The above challenges outline the main issues that operators need to work out to fulfill the promise of E2E network slicing, in 5G and beyond. However, solving these issues towards this ultimate goal may require years, specially for the first issue (i.e., the different slicing readiness across the different domains). In the meantime, operators need to look for workarounds to start commercializing and monetizing network slicing, incorporating solutions in their portfolio according to the set of slicing capabilities available in their networks by then. The population of the portfolio is not an easy task, given the quite fragmented landscape in standards and literature, with plenty of ad-hoc solutions that cover particular slicing aspects from different domains and under different assumptions. Defining a network slicing rollout plan based on the current collection of solutions is a critical activity for operators to succeed in the market. This activity consists of two steps. First, identifying all relevant solutions and positioning them in a common space, with multiple dimensions that reflect the E2E conception of network slicing. Secondly, defining a go-to-market strategy [3], based on deciding which solutions will be made available, when, for which customers, and under which business models.

The purpose of this paper is to address the first step, outlining a technology radar to model this common space. This radar presents a phased-based vision for the introduction of network slicing capabilities in commercial networks, considering all the domains impacted in operator assets, including the main three technology domains and the OSS. In this vision, the radar identifies different solutions for network slicing and captures them into four rings, each corresponding to a different timeline: as-is ring (today’s slicing), deploy ring (short-term slicing), test ring (medium-term slicing) and explore ring (long-term slicing). The position of each solution in the radar is done according to three different criteria: (i) the technology maturity of the solution, which is related to the readiness of the corresponding standards; (ii) the roadmap of commercial products, which specifies when the features associated with the solution will be available; and (iii) the relevance for the customers, which determines the prioritization of the solution over others.

To the best of our knowledge, this is the first work in the literature that provides a radar for E2E network slicing, with a focus on the rollout of this technology in carrier networks. The radar captures a complete landscape of network slicing solutions, linking them to different timelines. In addition to this timing, the radar will also outline the dimensions impacting slice realization, from E2E viewpoint. These dimensions are to be analyzed in each of the operator managed domains, including Radio Access Network (RAN), Core Network (CN), Transport Network (TN) and OSS. In the *RAN domain*, network solutions are to be discussed based on three dimensions: functionality (e.g., disaggregation and O-RAN integration), radio resource allocation and penetration (in micro and macro cells). The *CN domain* will focus on how to use and combine core network functions for different slices, including baseline and value-added functions, depending on isolation and customer requirements. In the *TN domain*, slicing is to be discussed based on the availability of transport technologies and SDN-enabled capabilities, including programmability and automation. Finally, in the *OSS domain*, aspects related to network slice lifecycle management and capability exposure (i.e., to expose slicing capabilities to customers through service APIs) will be taken into account. These dimensions are used to characterize the different solutions captured in the radar, providing guidance on how and where using them. This information, together with the timeline provided for these solutions, is the input material that enables an operator to define the plan for network slicing rollout. For further details on how to design and execute this plan, see recommendations reported in [4,5,6].

This article is structured as follows. Section 2 provides the technical background of E2E network slicing, with focus on the modelling, system architecture and deployment related aspects. Section 3 outlines the impact that the network slicing may introduce on the different technology domains. The understanding of these features will enable the reader to understand the radar, which is introduced in Section 4. The radar is the core contribution of this article, and hence deserves a detailed discussion, with a thorough analysis of all the solutions along the different dimensions: CN (Section 5), RAN (Section 6), TN (Section 7) and OSS (Section 8). Finally, Section 9 summarizes the main conclusions of this work.

## 2. Network Slicing: Concept, System Architecture and Deployment

This section provides a technical background of network slicing, outlining the main artifacts involved in their realization.

### 2.1. Network Slice Concept

A network slice provides a service-tailored connectivity pipe to one or more service applications hosted by the Data Network (DN). Examples of service applications include Immersive Reality (XR) streamers, IoT platforms or V2X backend servers. These applications can be associated with operator services (e.g., communication services) or with third party services. Devices subscribed to one service can establish communication with the service applications through the corresponding network slice, which will provide an enhanced connectivity profile in terms of functionality, performance and/or security [2].

The fact that makes network slicing an E2E concept is that the device-to-application connectivity pipe involves all the technical domains within the operator’s managed network, including the RAN, CN and TN domains.

The **RAN domain** allows connecting the end devices to the operator’s network using a wide variety of access technologies. In this paper, we focus on the Next Generation RAN (NG-RAN) [7,8]. The NG-RAN consists of multiple gNBs, which provide connectivity towards end devices using 5G New Radio (NR) technology. To take advantage of the benefits that RAN virtualization brings in terms of scalability and centralization, the standards have moved to a new architecture model where a gNB can be logically split into three entities denoted as radio unit (RU), distributed unit (DU) and centralized unit (CU). The NR protocol functions that correspond to each of these entities are determined by the so-called split options. Though there exist up to eight split options available for this gNB decomposition, after a thorough analysis the industry has opted for two: split 2, defined by the 3GPP and acting as a high layer split; and split 7-2x, defined by the O-RAN Alliance and acting as a low layer split [9]. The figure in Section 2.2 details the partitioning of NR protocol functions into RU, DU and CU, according to these two split options.

The **CN domain** allows end devices to send/receive mobile traffic to/from DN hosted applications or the Internet. In 5G, this functionality is provided by the 5G Core (5GC) [10]. Designed from its inception to be cloud-native, the 5GC follows a service based architecture (SBA), with the definition of a disaggregated and modular, containerized control plane which is fully decoupled from User Plane Functions (UPFs).

Finally, the **TN domain** is in charge of providing infrastructure connectivity between the RU (the entry point to the network for the device) and the DN (where the service applications are hosted). To that end, it makes use of a wide variety of forwarding devices, which are founded on different technologies (e.g., IP/MPLS, optical/DWDM and microwave/backhaul) and connected forming different topologies (e.g., ring, mesh, hub-and-spoke), across different aggregation levels. The TN domain sets up the data path across the different RAN and CN functions, by mapping their interfaces into Wide Area Network (WAN) infrastructure resources. According to this mapping, different TN segments can be outlined:

- Fronthaul segment, scoping the data path between the RU and the DU. This data path implements the O-RAN fronthaul interface (split 7-2x).The control, data, management and synchronization planes of this interface are defined in [11,12].
- Midhaul segment, which sets up the data path between the DU and the CU. This data path implements the 3GPP F1 interface (split 2) [13].
- Backhaul segment, established between the CU and the UPF. It covers two 3GPP interfaces: N3 (CU-to-UPF) and N9 interface (UPF-to-UPF). When the UPF connected to the CU is the anchor UPF, then the N9 interface is not needed [10].
- DN segment, establishing connectivity between the (anchor) UPF [10] and the DN. This segment is the transport level realization of the 3GPP N6 interface [14].

To make slicing a reality, every technical domain is split into one or more logical network partitions, each referred to as a network slice subnet. The definition of multiple slice subnets on a single domain allows this segment to provide differentiated behaviors, in terms of functionality and/or performance. The stitching of slice subnets across the RAN, CN and TN results in the definition of network slices.

**Figure 1:**
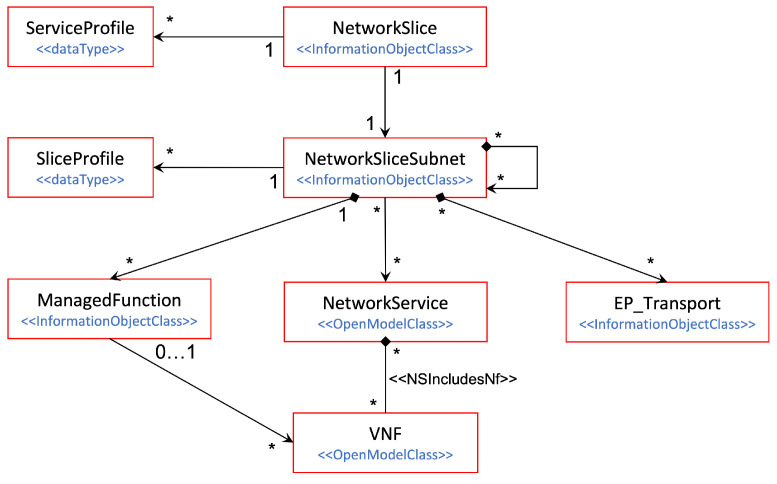
3GPP Information Model of a network slice: the Network Slice NRM fragment.

The rules for the definition of network slice subnets and their composition into network slices are detailed in the 5G Network Resource Model (NRM), specifically in the Network Slice NRM fragment [15]. This fragment captures the information model of 5G network slicing. As seen in Figure 1, this model specifies the relationships across the manageable entities, each represented as a separate Information Object Class (IOC). An IOC captures the semantics and attributes of a manageable entity; in other words, it defines the class based on which instances (objects) from this entity can be created. In the model, we have four different IOCs: (i) *NetworkSlice IOC*, representing a network slice; (ii) *NetworkSliceSubnet IOC*, associated with a network slice subnet; (iii) *ManagedFunction IOC*, which represents a 5G network function; and (iv) *EP_Transport IOC*, which represents an interface associated with transport level information, e.g., transport address, reachability information, and QoS profiles. Note that for NetworkSlice and NetworkSliceSubnet IOCs, two additional constructions are defined:

- ServiceProfile: represents the requirements that the slice needs to support for a particular service. The 1:N relationship of this construction with the NetworkSlice IOC is because one network slice can host multiple services, as long as they do not impose conflicting requirements. These services can be from the same customer (the slice is dedicated for this customer) or different customers (the slice is used for serving multiple customers).
- SliceProfile: similar to the ServiceProfile, but applied to the slice subnet level.

Though multiple associations can be found across these IOCs, the most typical case consists in having one slice consisting of two slice subnets: one including NG-RAN functions (RAN slice subnet) and the other 5GC functions (CN slice subnet). Each network slice subnet can be deployed as an ETSI network service (via the *NetworkService* class), provided that one of the network functions is realized as a Virtualized Network Function (VNF) [16]. Finally, the EP_Transport IOC features the TN slicing behavior across the RAN and CN slice subnets, by mapping the QoS requirements associated to the different interfaces (e.g., F1, N3, N6, etc.) into appropriate WAN resources.

### 2.2. Architectural Framework for Network Slicing

**Figure 2:**
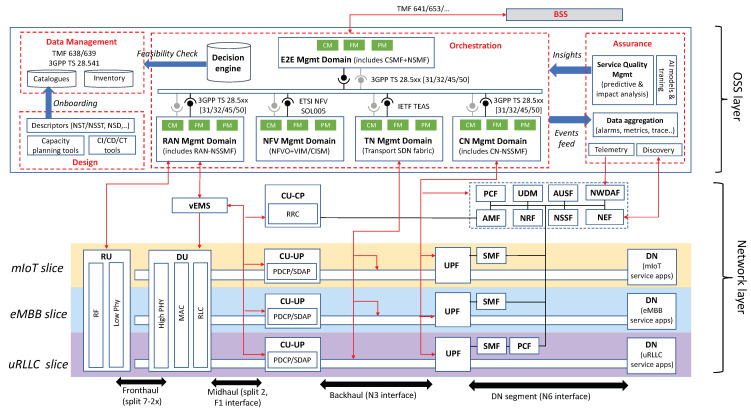
Network slicing system architecture.

Figure 2 illustrates the system architecture design for network slicing. This system is structured into two layers: the *network layer*, which provides the individual slices with the required user and control plane functionality, across all technical domains; and the *OSS layer*, which hosts all the assets that are used for the design, provisioning and operation of network slices.

The network layer is formed of a collection of modular network functions that can be flexibly combined together to build up network slices. Figure 2 shows an example with three different slices, one for each main 5G service category. The fact that every slice needs to be provisioned with a service-tailored user and control planes justifies the allocation of dedicated NR and 5GC network functions in their RAN and CN slice subnets. Which network functions are to be dedicated per slice and which ones can be shared with other slices needs to be analyzed case by case, as it depends (i) on the isolation requirements of the slice under consideration, and (ii) the type of customer that will consume this slice. Further discussion on this topic is captured in Section 5.1 and Section 6.1.

The OSS layer conveys all the Operation, Administration and Maintenance (OAM) tools that operators may use to manage the different slices across their entire lifetime [17]. These tools are classified into four main groups, depending on their scoped functionality: (i) design, (ii) data management, (iii) assurance and (iv) orchestration. The most notable group is the orchestration, responsible for all the activities related to slice provisioning (i.e., going from a service order to a deployed network slice) and slice operation (i.e., keep the deployed slice at the desired state at run-time). This collection of activities shall be performed consistently across all the technical domains, with an E2E perspective. The specificities of these domains, each with a different pace of technological evolution and with legacy from multiple vendors, unveils non-negligible integration issues for operators. This is exacerbated as the number of slices running in parallel increases.

To cope with the above integration and scalability challenges, operators are required to adopt novel architecture approaches on the orchestration group. Service-based paradigm, which is about designing software architectures using Application Programming Interfaces (APIs) based on web-based technology, is considered as a potential facilitator in this respect. Originally conceived for 5GC, this architectural style can also be applied to the OSS layer, resulting in a Service-Based Management Architecture (SBMA). The SBMA consists of replacing traditional management entities (e.g., Network Managers) with a federated set of management functions that provide services to each other using REST APIs. The adoption of SBMA allows fleeing from point-to-point protocol interfaces (e.g., 3GPP Itf-N interfaces) to a service bus that interconnects all the management functions and polices the interactions across them. Different SDOs have already captured the benefits of having a SBMA in their architecture specifications. For example, 3GPP SA5 [18] and ETSI ISG ZSM [19] have defined their architectural frameworks based on SBMA. Even ETSI ISG NFV, which originally chose an interface-centric approach for the design of the Management and Orchestration (MANO) framework, has now decided to migrate towards a SBMA from NFV Release FOUR on wards [20].

As seen in Figure 2, the management functions building up the OSS’s orchestration group are arranged into five separate domains: RAN, NFV, TN, CN and E2E management domains. This design criterion represents a separation of concerns that is reasonable from the operator’s viewpoint, and which relies on two principles:

- The independent management of network resources and functions from different technical domains. This facilitates a decoupled evolution of RAN, CN and TN, and allows the operator to select the technologies and vendor solutions they want for every technical domain.
- A clear separation between management (i.e., OAM activities on individual technical domains) and orchestration (i.e., coordination and conflict resolution activities across technical domains). In the proposed solutions, the RAN, CN and TN management domains are focused on management activities, while the NFV and E2E management domains are the ones responsible for orchestration.

The interactions across the different management domains are done with a service bus, which features the ZSM cross-domain integration fabric [19]. As seen, it is important for the different domains to make capabilities available for external consumption through standard APIs. Figure 2 captures relevant references for these APIs.

### 2.3. Network Slice Description

One of the main business cases for network slicing is Network Slice as a Service (NSaaS) [17]. In this business model, an industry vertical (acting as the network slice customer) requests the network operator (acting as the network slice provider) to allocate a dedicated network slice satisfying a particular set of service requirements. With a large variety of emerging verticals in the market, it is fundamental for the operator to define a unified ability to interpret service requirements from different verticals, and to represent them in a common language. This unification will help the operator capture vertical-specific service requirements and translate them into appropriate network slice provisioning actions.

In this regard, the GSM Alliance (GSMA) has promoted the idea of having a universal slice blueprint providing a point of convergence between telco and vertical industries on network slicing understanding. This blueprint, known as the Generic network Slice Template (GST) [21], contains a set of attributes that allow the characterization of any network slice. The most representative GST attributes are included in Table 1.

A Network Slice Type (NEST) is the result of filling GST attributes with values according to the service requirements. In essence, a NEST is a filled-in version of a GST, and can be used by an operator and a vertical customer to agree on the Service Level Agreement (SLA). Different NESTs allow the description of different network slices. For slices based on 3GPP 5G service categories, the operator may have a set of standardized NESTs (S-NESTs). For slices addressing specific industry use cases, the operator can define additional NESTs (P-NESTs) [22].

Table 1 provides one example with three NESTs, one for each slice represented in Figure 2. The table also qualifies the impact of the NEST attributes on every technical domain.

### 2.4. From a Service Order to a Deployed Network Slice

For enabling NSaaS, the operator registers in their portfolio a collection of service offerings, each representing a slice associated with a SLA. This SLA includes two main types of information: (i) technical information, which is captured in a NEST; and (ii) charging and pricing information. For the request of a network slice, the vertical customer browses the operator’s portfolio, selects the service offering that best fits their needs, and issues the corresponding service order. From this point on, the following activities are triggered on the operator side:

1. The operator’s BSS (Business Support System) captures the service order. It uses the charging and pricing information to configure the customer profile, and forwards the technical information (the NEST) to the E2E management domain using TM Forum Service Ordering API [23].
2. In the E2E management domain, the Communication Service Management Function (CSMF) translates the NEST parameter values into the ServiceProfile construction (see Figure 1).
3. The CSMF requests the allocation of a network slice based on this ServiceProfile. The CSMF sends this request to the Network Slice Management Function (NSMF), using the *allocateNsi* operation (see clause 6.5.1 from 3GPP TS 28.531 [24]).
4. With the network slice allocation request, the NSMF is asked to deploy a network slice instance (NSI) on the operator’s managed network infrastructure, in such a way that the service requirements captured in the ServiceProfile are fulfilled. Before beginning the deployment of network slice subnet instances (NSSIs) and the reservation of WAN resources across them, the NSMF shall make sure that the network slice allocation is feasible. To that end, it requests the Decision Engine (see Figure 2) to perform a feasibility check procedure. The complete procedure execution can be separated into two parts. The first part checks for the qualitative network capabilities that the network slice instance requires, e.g., availability of a specific radio access technology or feasible network function configurations. This is expected to be completed rather quickly and can therefore provide a quick reply in the case of a negative (“network slice instance unfeasible”) response. In case of a positive qualitative check, the second part quantitatively checks if there are enough infrastructure resources (including radio, WAN and compute resources) available for use. It also calculates confidence values if resource availability is associated with statistical uncertainty, e.g., due to statistical fluctuations in resource consumption of already deployed slice instances.
5. If feasible, the NSMF proceeds with the NSI allocation, based on the allocation of (i) the RAN NSSI, (ii) the CN NSSI, and (iii) the WAN resources providing end-to-end connectivity. In this process, the NSMF interacts with the Network Slice Subnet Management Functions (NSSMFs) from the RAN and CN management domains, and with the SDN fabric from the TN management domain. The NSSMFs may interact in turn with the NFV Orchestrator (NFVO) through SOL005 [25], for the cases where the NSSIs can be deployed as ETSI network services.

**Figure 3:**
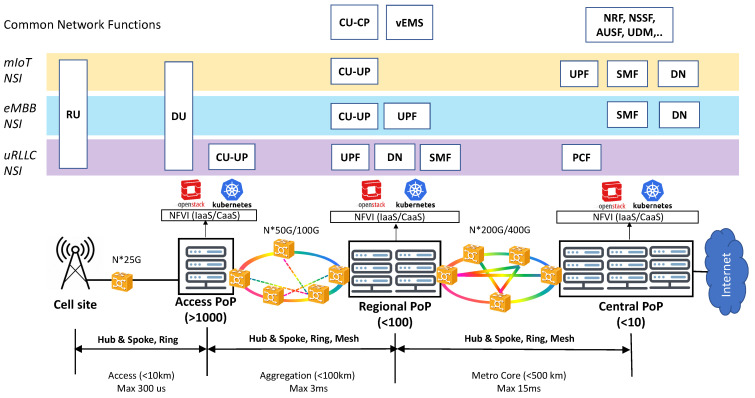
On the deployment of network slices.

Figure 3 illustrates the deployment view of the network slices shown in Figure 2. This view shows these slices are allocated on the operator’s managed infrastructure, in the form of NSIs. For this allocation, it is assumed that (i) the network slices have been ordered according to the NESTs specified in Table 1, and (ii) the infrastructure consists of a RAN with cell sites attached via dedicated fibers to a three-tier TN. This capillarity in TN design allows the distribution of compute capacity, across Points of Presence (PoPs), which are physically deployed at three different aggregation levels:

- Central PoPs, which correspond to large-scale core cloud sites. They are typically built with commodity (x86 or ARM based) hardware, and are ideal to host IT applications and delay-tolerant telco workloads.
- Regional PoPs, which represent Central Offices featuring the telco edge cloud [26]. The regional PoPs provide virtualization capabilities closer to service delivery endpoints in order to reduce the delay budget, making them ideal to host delay-critical telco workloads.
- Finally, access PoPs, which are associated with far edge sites. Much more distributed and closer to cell sites than regional PoPs, the access PoPs provide execution environments for hosting workloads with real-time requirements, e.g., virtualized DU instances (vDUs). In this regard, commodity hardware is no longer valid; they need to be equipped with advanced, rich-featured CPU architectures (e.g., Intel Xeon) and hardware acceleration solutions (e.g., FPGA, structured ASICs, etc.) instead [27].

## 3. Impact of Network Slicing

The introduction of slicing will impact all the technical domains of the network. In this section, we review the features required on these domains to support slicing. These constitute the basis for the understanding of the different solutions that will be explained later on, in Section 5, Section 6, Section 7 and Section 8.

### 3.1. CN Slicing

The impact of network slicing in the CN domain can be summarized into three main topics: slice identity management, slice-aware device connectivity and the allocation of separate 5GC functions.

#### 3.1.1. Slice Identity Management

The network slicing feature was first introduced in Release 15, with the ability of the 5GC to support multiple network slices and differentiate among them. This differentiation is done using two signalling identifiers: the Single Network Slice Selection Assistance Information (S-NSSAI) and the Network Slice Selection Assistance Information (NSSAI).

The S-NSSAI identifies a network slice across the UE, RAN and the 5GC. It is a 32-bit parameter comprised of two fields:

- A Slice/Service Type (SST): mandatory 8-bit field that refers to the expected network slice behavior in terms of features and supported services. The SST field may have standardized and operator-specific (non-standardized) values. The standardized SST range [10] includes values from 0 to 127, while values 128 to 255 belong to the operator specific range. For now, the following SST values have become normative: SST = 1 (enhanced Mobile Broadband), eMBB), SST = 2 (Ultra Reliable Low Latency Communication, uRLLC), SST = 3 (massive IoT, mIoT), SST = 4 (Vehicle to Everything, V2X) and SST = 5 ( High-Performance Machine-Type Communications, HMTC).
- A Slice Differentiator (SD): optional 24-bit field that allows the operator to differentiate among multiple network slices with the same SST. This differentiation can be in terms of slice features (e.g., mobile vs fixed-wireless access services, charging), customer information (tenancy) and slice priority.

An NSSAI is a collection of S-NSSAIs sent by the device to assist the network in selecting a particular network slice for this UE. Within the Public Land Mobile Network (PLMN), the NSSAI is managed at the Tracking Area level in the RAN, and at the Registration Area level in the 5GC. Different types of NSSAIs exist, including Configured NSSAI (NSSAI provisioned in the device), Subscribed NSSAI (NSSAI stored in the UDM), Requested NSSAI (provided by the UE to the serving PLMN during registration) and Allowed NSSAI (provided by the serving PLMN to the UE during registration) [10]. The 5GC uses the Requested NSSAI for slice selection and validation, and returns the Allowed NSSAI. The Allowed NSSAI indicates the S-NSSAI values that the UE can use in the serving PLMN for the current Registration Area.

**Figure 4:**
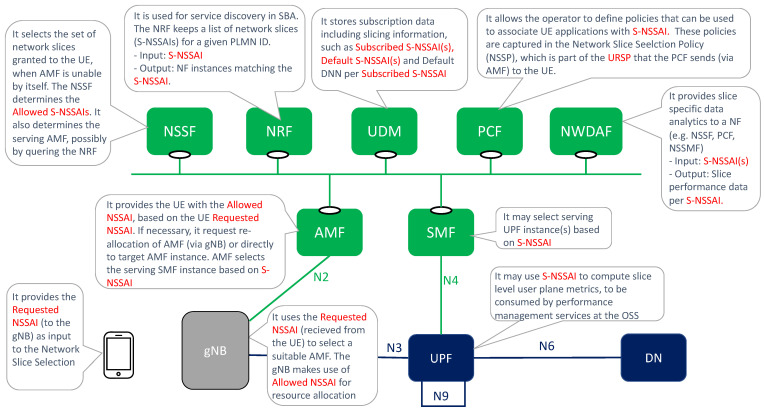
Impact of slicing in 5GC.

Figure 4 summarizes the use of S-NSSAI and NSSAI artifacts in the 5G network.

#### 3.1.2. Slice-Aware Device Connectivity

According to 3GPP specifications, the Allowed NSSAI can include a maximum of eight S-NSSAI values [10]. This means that a device can establish Packet Data Unit (PDU) sessions with up to eight slices at the same time.

The device can have different client applications (e.g., internet browsing applications, enterprise applications, XR applications), each requiring the connection to a different slice. To make this possible, the device needs to be made slicing aware, something that is achieved with the introduction of the UE Resource Selection Policy (URSP) [28]. The URSP is a network slicing feature enabled by the Policy Control Function (PCF), which informs the network slice status to the UE via the Access and Mobility management Function (AMF). It is composed of a number of URSP rules that map application information (e.g., client application ID, device Operation System ID, IP descriptors) with network slice information (e.g., S-NSSAI, Session and Service Continuity, Data Network Name). The device uses the URSP to determine which PDU session shall be chosen for a particular application based on URSP rules. For further information on the URSP and its use for slicing support at the device side, please see [29].

#### 3.1.3. 5GC Network Functions

As commented in Section 2.2, the allocation of dedicated network functions on a network slice allows it to be tailored to the specific needs of hosted service(s). Where there is more potential to making this customization is on the 5GC side.

It is not the goal of this subsection to discuss which 5GC functions are to be dedicated to a slice; indeed, as we will see in Section 5.1, this entirely depends on the business requirements of individual customers. The purpose of this section is instead to outline the importance of some 5GC functions when building up CN slice subnets. In this regard, the UPF is the most valuable network function to be dedicated, followed by control plane network functions (SBA).

The importance of having a dedicated UPF comes from two important reasons: (i) a tailored user plane QoS, and (ii) an improved availability and reliability. The first point refers to the ability of allocating an UPF with required resource capacity where needed, e.g., close to customer premises to ensure low latency. The second point means that having a dedicated UPF instance allows optimal redundancy level to be achieved, and the risk of service interruption for the slice to be reduced, ensuring that established sessions can survive for a period of time, even when the connection to the control plane functions is lost.

The control plane functions are the second-most valuable assets to dedicate. For example, with a dedicated Session Management Function (SMF) [10], it is possible to make changes to established sessions and establish new sessions for a period of time, even if the connection to UPFs is lost.

### 3.2. RAN Slicing

In the NG-RAN, the introduction of slicing has an impact on three main aspects: gNB configuration, mobility support and Radio Resource Management (RRM) procedures.

#### 3.2.1. gNB Configuration

A gNB can be configured to support multiple slices. This configuration, done via the NSSMF, is based on the following principles:

(a) Network slices are defined within a PLMN. In RAN sharing scenarios, where multiple PLMNs share the same cell, each operator needs to link S-NSSAIs with the PLMN ID.
(b) The gNB serves a cell. The cell belongs to a tracking area, which is identified with two artifacts: Tracking Area Code (TAC), i.e., local identifier, and Tracking Area Identifier (TAI), i.e., universal identifier. The TAI is a {PLMN ID, TAC} tuple, and it is relevant in RAN sharing scenarios. To indicate the tracking area to which the cell belongs to, the gNB broadcasts one or more TAIs, i.e. one TAI per hosted PLMN ID [30].
(c) A network slice is linked to a tracking area. This is because S-NSSAIs are managed per tracking area [31].

Based on the above principles, it can be noticed that all cells belonging to the same tracking areas must serve the same set of network slices. Once the gNB is set with supported slices (per TAI), its mission is to map traffic from individual PDU sessions into appropriate NG-RAN resources. This is done by associating the tuple {S-NSSAI, PLMN ID} with one Dedicated Radio Bearer (DRB). The profile of the DRB is configured with RRM parameters which are tailored to the service requirements of the slice.

#### 3.2.2. Mobility Support

When the device moves from one cell to another, a handover procedure is triggered. The handover request (from the source gNB to the target gNB) includes the network slices assigned to the UE, specifying the tuple {S-NSSAI, PLMN ID} for each active PDU session. According to the principles listed earlier, it is clear that handover requests between gNBs from the same tracking area are always successful. However, in the case of mobility outside a tracking area, it might happen that one or more PDU sessions could not be transferred, because the associated S-NSSAI are not available in the target cell. In traditional radio control admission solutions, where handover acceptance is subject to the admission of all radio bearers, this scenario would result in an automatic handover rejection. To solve this all-or-nothing approach, partial admission control mechanisms are being developed. These mechanisms allow the admission of those PDU sessions whose associated S-NSSAIs are supported in the target gNB. The logic is as follows:

- The target gNB will send handover request ACK with Admitted PDU session and Not Admitted PDU session.
- If all the S-NSSAIs in the handover request are not admitted, the handover will be rejected.

**Figure 5:**
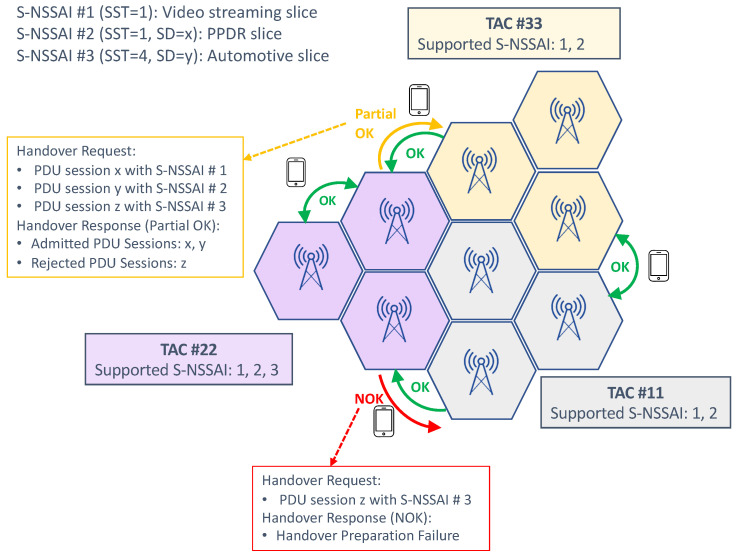
Slice-aware mobility.

Figure 5 shows an example of mobility support using this partial admission control for handover management.

#### 3.2.3. RRM Procedures

The gNB includes a set of RRM procedures that govern the allocation of NR cell resources across existing slices, in such a way that shortage of resources in one slice does not break the SLA of another slice. There are two fundamental RRM procedures: admission control and scheduling.

The task of admission control is to admit or reject the establishment requests for new radio bearers. Admission control can be based on number of users (RRC connections) or number of DRBs. The first option allows limiting the number of UEs accessing a specific slice based on SLA requirements. The second option is based on reserving enough DRBs for each slice, according to their estimated data volume.

The scheduling allows the gNB to dispatch available Physical Radio Blocks (PRBs), i.e., frequency-time resource grids, across the different slices, in such a way that the QoS requirements associated with their PDU sessions can be fulfilled. These requirements are expressed with the 3GPP 5G Quality Indicator (5QI) [10].

At a very high load, admission control provides the scheduler with sufficient resources to secure QoS of all the admitted users. To that end, it is very important to design an efficient admission control algorithm that can take into account the overall resource situation, the priorities of users based on their category level, the QoS of the in-progress request and the QoS requirements of the new radio bearer requests.

**Figure 6:**
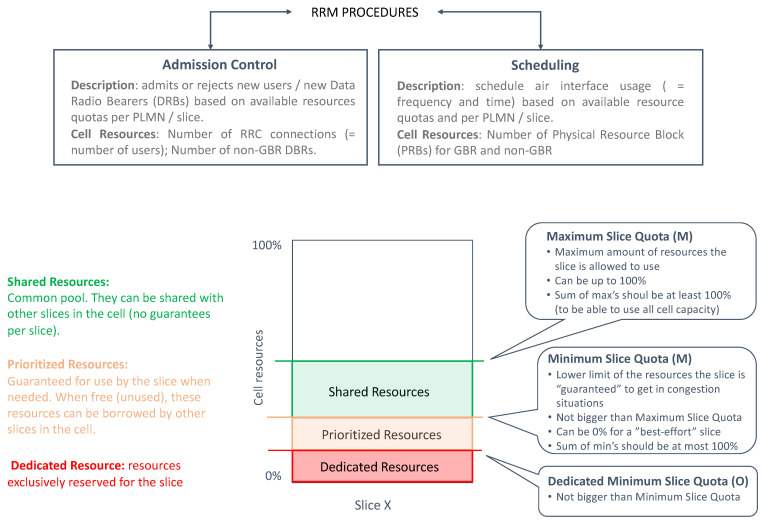
RRM procedures for slicing.

The operation in the admission control and scheduling procedures is, in both cases, based on the configuration of the following per slice quotas: dedicated minimum slice quota (optional), minimum slice quota (mandatory) and maximum slice quota (mandatory). Figure 6 provides a summary of these three quotas. These quotas need to be specified for each RRM procedure, since the managed NR cell resources are different:

- For admission control, the NR cell resources correspond to either RRC connections (option 1) or DRBs (option 2). For option 1, it is the CU-CP which configures the quotas. For option 2, it is the CU-UP.
- When scheduling, the NR cell resources correspond to PRBs, based on which per slice quotas are defined.

In this work, we will focus on scheduling aspects. Table 2 shows different examples on how to configure the slice quotas for scheduling. As seen, depending on the values set for these quotas, the slice can be profiled into different categories.

### 3.3. TN Slicing

Unlike the NG-RAN and 5GC, the TN domain is out of the scope of the 3GPP network slice concept. 3GPP provides slicing solutions for the RAN and CN domain, but not for the TN. However, to maintain consistency on the slice established between the device and the service application, there is a need to map 3GPP slice criteria into appropriate transport capabilities offered in the fronthaul, midhaul, backhaul and DN segments. This is not trivial.

On the one hand, there is the need to configure WAN resources in such a way that the requirements captured in the ServiceProfile and SliceProfile can be fulfilled in the TN substrate. This requires translating network function layer requirements associated with S-NSSAI information (e.g., maximum delay budget, data rates, availability, mobility speed, usage density) into transport network characteristics that include bandwidth, latency and criteria such as traffic prioritization, directionality, protection and disjoint routes. This translation is done at provisioning time.

On the other hand, there exists a wide availability of transport technologies in carrier networks. These technologies provide multi-layer connectivity services using different topologies (e.g., hub-and-spoke, ring, point-to-point, point-to-multipoint). Though they do not support slicing natively, these technologies are able to mimic slicing behavior, if configured (and combined) properly.

In this section, we focus on the main enablers for these two open questions.

#### 3.3.1. On the Mapping of 3GPP Slice Information into TN Nodes

**Figure 7:**
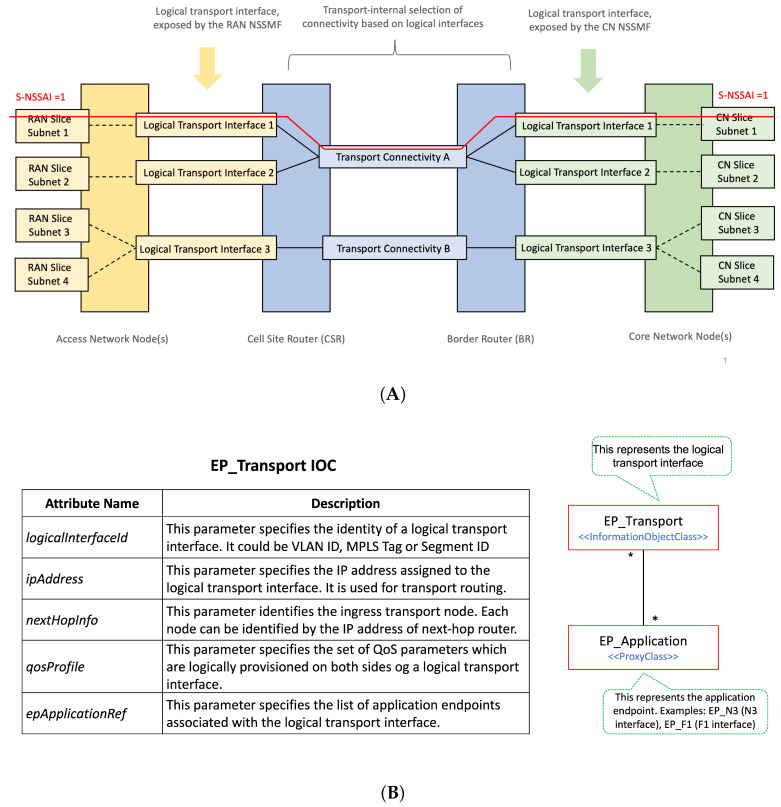
On the use of logical transport interfaces for TN slicing support. (**A**) Traffic segregation and mapping to S-NSSAIs; the BR is also referred to as Provider Edge (PE) router. (**B**) Schema for the EP_Transport IOC.

To configure the TN slicing behaviour in the WAN resources, the TN management domain needs the following information:

- Network slice topology. The TN management domain needs to know the application endpoints of the slice to determine the needed WAN resources, which are either physical or virtual nodes. NSMF/NSSMFs provide the application endpoints [32] of 3GPP network functions taking part in the RAN and CN slice subnets and, if applicable, further information such as the next-hop router IP address configured in these network slice subnets. For example, the CU-UP application endpoints are the IP addresses/VLAN IDs associated with the F1 and N3 interfaces. The TN management domain correlates this information with the transport network topology and derives the (cell site or border) routers connecting to network functions.
- Traffic segregation and mapping to S-NSSAIs. As 3GPP network functions can be shared by multiple network slices, it is necessary to segregate traffic belonging to specific slices on transport interfaces. One option for traffic segregation is to assign application endpoints to a specific set of S-NSSAI values. This solution is rather simple, as the TN can map packets to connectivity services based on application endpoints, provided that (i) the allocation of S-NSSAI to endpoints is known, and (ii) the application endpoints are visible on the transport layer. While this is the simplest solution in many cases, it is not a universal solution, as the application endpoint addresses are not always visible to the site router, e.g., when there is encryption using IPSec. An alternative solution is the concept of logical transport interfaces, as shown in Figure 7A. A logical transport interface is a virtual interface separated from application endpoints. It can be, for example, a specific IP address/VLAN combination corresponding to an IPSec termination point, or an identifier (e.g., MPLS label, segment ID) that the TN recognizes, or it can be just a logical interface defined on top of a physical transport interface. As long as the interface identity can be derived from packet headers, the TN nodes can perform the mapping to transport connectivity services.
- Reachability information. Each logical transport interface carries the traffic associated with some application endpoints that may be using IP addresses separate from the transport interface. These IP addresses must be reachable, hence they need to be advertised to populate forwarding tables. A 3GPP network function can advertise such reachability information by running a dynamic routing protocol towards the next hop router.
- QoS requirements. To satisfy the service requirements captured in ServiceProfile and SliceProfile, each logical transport interface needs to be bound to a QoS profile that includes the applicability and use of DiffServ Code Points (DSCP) [33] and QoS related properties on that interface.

To allow the TN management domain to receive this information from the 3GPP management system, the EP_Transport IOC [15] is defined. Part of the Network Slice NRM fragment (see Figure 1), this class allows the capture of the information that shall be exchanged between the 3GPP management system (E2E management domain, RAN management domain and CN management domain) and the TN management domain. This information is used to configure WAN resources in such a way that the requirements captured in ServiceProfile and SliceProfile can be fulfilled. Figure 7B shows the construction of the EP_Transport IOC, and how it maps the logical transport interface to application endpoints. Notice that one EP_Transport (representing a logical transport interface) can be associated with more than one multiple EP_Application (representing an application endpoint of a 3GPP network function), but also the other way around. While the first case captures the typical situation, the second case can be used for the sake of resilience or load balance in the TN. For example, in Figure 7A, instead of configuring multiple nextHops for one EP_Transport to allow multiple optional “links” between the gNB port and the cell site router, the solution adopted is as follows: to configure one nextHop for each EP_Transport, but have more than one EP_Transport for an EP_N3 to achieve similar load balance or resilience goal.

#### 3.3.2. Transport Technologies

To convey slice traffic in the transport network, multiple forwarding plane technologies can be used. Depending on their isolation capabilities, these technologies can be clustered into two main categories: soft slicing (the traffic loading on one slide may degrade the performance of other slices, because of the use of statistical multiplexing and service classes) and hard slicing (the traffic loading on one slice has no impact on the traffic from any other slice, including QoS effects).

The soft slicing category uses packet-based technologies to provide traffic-engineered and traffic-managed isolation of resources. This category encompasses Layer 2 and Layer 3 technologies, including tunnelling (e.g., VxLAN, MPLS) and virtualization (e.g., VPN, VLAN) based technologies. The mutual impact of QoS of slices sharing the same infrastructure resources may be mitigated by traffic engineering including, for example, limiting the statistical multiplexing ratio, or traffic policing on each network slice.

Hard slicing can be guaranteed through independent circuit switched connections (e.g., dedicated wavelength, dedicated TDM time slot) for the exclusive use of a single network slice. Unlike soft slicing, this category is thought of as being implemented at Layer 1, using techniques much more closely coupled to the hardware itself, such as optical transport network switching [34] or novel Ethernet-based solutions like Flex-Ethernet [35,36].

**Figure 8:**
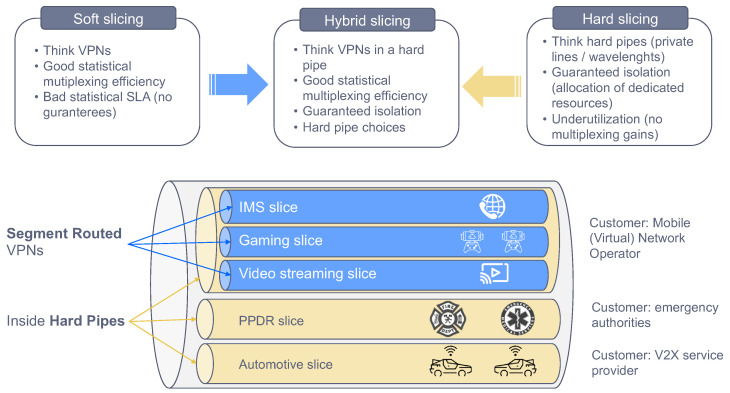
Soft, hard and hybrid slicing in transport networks.

Depending on the customer requirements, the operator may go for soft slicing or hard slicing, or a mixture of the two. Indeed, it is possible to combine them as shown in Figure 8, with hard slicing ensuring a dedicated capacity chunk for the customer, and soft slicing providing traffic seggregation among the services belonging to this customer. This approach preserves a cost-efficient solution to the customer that has multiple services, and wants them not to be impacted with traffic congestion or faults issued by services from other customers.

## 4. Network Slicing Technology Radar

Network slicing is an E2E solution, covering the three technology domains: RAN, CN and TN. The provisioning and operation of the different slices, and the lifecycle management of hosted services, is done with a cloud-native OSS stack composed of a number of management domains, including vertical domains (e.g., RAN, CN, TN management domains) and horizontal domains (e.g., MANO and E2E management domains). The maturity level of slicing varies across all these technology and management domains. This fact, together with the integration complexity of carrier networks (e.g., brownfield facilities, multi-vendor solutions), defines challenges that operators need to work out to enable the full promise of network slicing in a later phase.

Although standards and technologies do not currently support full slicing capabilities, it is important to get started with early-stage slicing and to use a vision of what is desired in the longer term to guide progress and focus. For operators, it is important to take a phased-based approach, establishing a process to incorporate learnings at each stage of the slicing journey.

**Figure 9:**
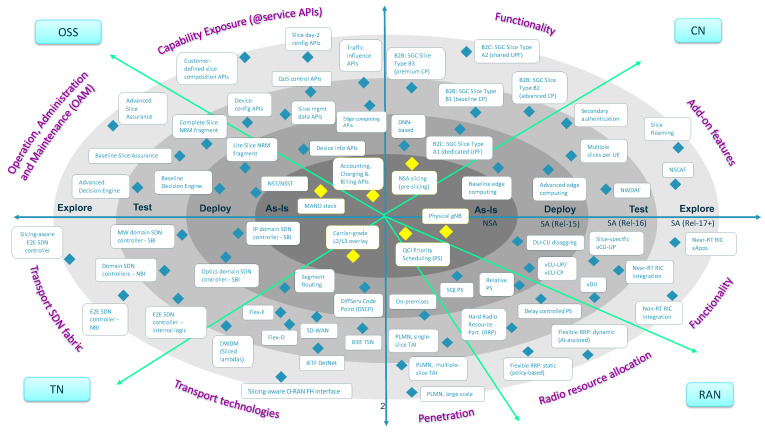
Network slicing technology radar.

The mission of this section (and upcoming ones) is to present a network slicing technology radar that can help operators to build their own journey towards the commercialization and monetization of slicing. As shown in Figure 9, this radar captures a list of solutions for network slicing impacting all relevant operator’s sub-systems, including RAN, TN, CN and OSS. This is complemented by an assessment work, called ring assignment. In particular, we use four rings with the following semantics:

- **As-is ring**: represents solutions that are available in today’s carrier networks. These solutions are typically associated with technologies that operators have high confidence in, with low risk and recommended to be available across the entire service footprint. In terms of 5G roll-out strategy, this corresponds to 5G NSA (Non Standalone) [37].
- **Deploy ring**: covers the slicing solutions that can be applied in early 5G SA (Standalone) networks, based on 3GPP Release 15 standards. Some operators have already started to activate their SA networks, while some others expect to get them operationally ready within next year. With this timing in mind, we can say that this ring captures proven slicing solutions that operators may integrate in the short-term.
- **Test ring**: captures slicing solutions that are much more focused on satisfying requirements from uRLLC and mIoT services. Associated with brand new Rel-16 features, these solutions have great potential but are unproven in production networks, hence it is worth operators investing in prototyping efforts in order to evaluate their performance and impact. This evaluation is typically done with commercial trials, either bilateral or multi-vendor, and different Proof of Concepts (PoCs). The upgrade towards Rel-16 is expected within the next 2–3 years; this means that test ring represents slicing solutions that might be available in the medium term.
- **Explore ring**: includes slicing solutions that are foreseen in the long run, starting in the next 4–5 years. These solutions, tied to features from 3GPP Rel-17 on wards, promise to provide great potential, though their impact and commercial availability is still far from crystal clear. The role of the operator is to keep track of their evolution through exploratory activities such as the ones done in research and innovation projects, e.g., 5G-VINNI [38], 5GROWTH [39] and 5G-CLARITY [40].

As outlined in Section 1, for the position of each solution into this radar, three criteria have been considered: (i) the technological maturity of the solution, which is subjected to the readiness of the standards; (ii) the roadmap of commercial products, which specifies when the features associated with the solution will be available; and (iii) the relevance for the customers, which determine the prioritization of the solution over others. The following sections provide details on these solutions, across the involved subsystems: CN (Section 5), RAN (Section 6), TN (Section 7) and OSS (Section 8).

## 5. CN Domain

In this domain, the technology radar captures information from two different dimensions: functionality and add-on features. The *functionality* dimension deals with the discussion on how to use CN functions for the construction of different network slices, scouting different deployment options for these slices depending on the isolation and business requirements of hosted services. On the other hand, the *add-on features* dimension refers to the set of value-added solutions that complement and extend baseline slice functionality. The network operator can optionally make use of these solutions to either (i) provision enriched services to the customer, i.e., new revenue streams; or (ii) streamline internal network operation, i.e., OPEX savings.

### 5.1. Functionality

**Figure 10:**
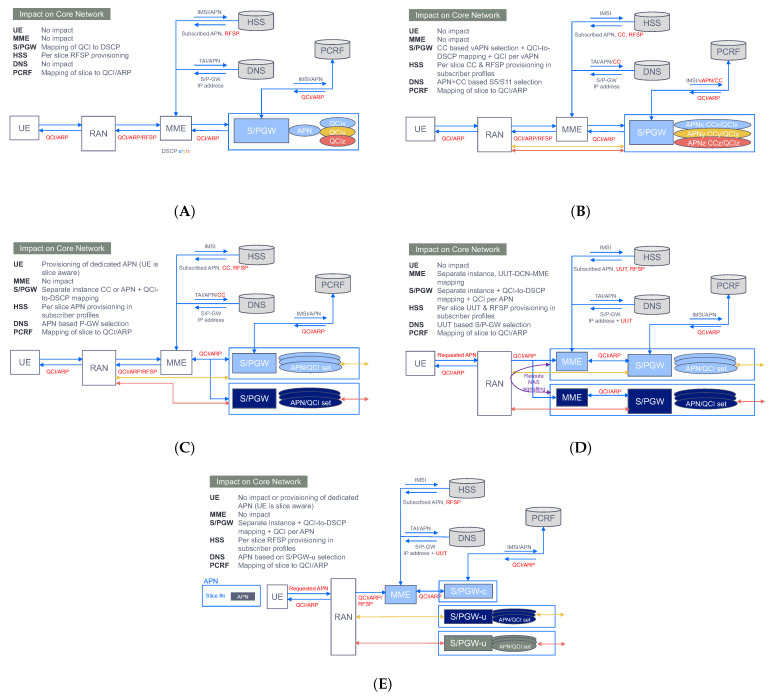
Different deployment options for NSA slicing. (**A**) QCI based common APN-S/PGW. (**B**) Virtual APN/QCI based on Charging Characteristics (CC). (**C**) Dedicated S/PGW. (**D**) DECOR. (**E**) Control User Plane Separation.

The first 5G commercial networks available worldwide are based on NSA. The reason is that most communication service providers are looking to deliver mainly high-speed connectivity to consumers already with 5G-enabled devices today. For these providers, the NSA mode makes the most sense, because it allows them to leverage their existing packet core assets (EPC) rather than deploy a completely new 5G network. In this mode, where 5GC does not exist, there is no native slicing support, as outlined in Section 3.1. However, this lack of slicing enablers (e.g., no S-NSSAI support) does not mean the operators are unable to provide service differentiation and traffic segregation at the core side; in fact, there are a number of solutions that allow the EPC to enforce some level of traffic separation for overload mitigation when having multiple services. Figure 10 depicts a functional description of some of these solutions, tagged with **NSA slicing (pre-slicing)** wording. As seen, the capabilities across these solutions are quite different, ranging from basic QoS differentiation (e.g., QCI based common APN-S/PGW) to a complete packet core separation (e.g., DECOR [41]), with a number of variants in between, some of them subjected to technology availability. For example, NFV technology is a must for the implementation of the control user plane separation solution (Figure 10E).

As communication service providers set their sights on new revenue streams from groundbreaking 5G services (i.e., uRLLC, mIoT, V2X services), they realize the need to migrate to the SA mode, which represents the target 5G system architecture [42]. With the first commercial 5GC solution suites already available in the market, operators can bring native slicing functionality into their carrier-class facilities. Despite being Rel-15 complaint, these first solution suites typically provide very limited capabilities in relation to S-NSSAI support; in fact, many of them offer single-slice support, with their AMF/SMF implementations only able to deal with one S-NSSAI at a time. The fact that one single slice can be configured in the 5GC prevents the operator from using separate slices to achieve service/customer traffic segregation. In these circumstances, non-NSSAI assisted solutions shall be used instead. One example is the provisioning of separate Data Network Name (DNN) [10] for different services/customers. This **DNN-based solution** is equivalent to the pre-slicing solution shown in Figure 10B, where virtual APN/QCI (EPC artifacts) are now replaced with DNN/5QI (5GC artifacts).

With the ever-increasing adoption of DevOps practices in the telco industry, 5GC software may be developed, delivered, tested and brought into operation incrementally at a far higher cadence than it was before. The CI/CD pipeline [43] will allow vendors to reduce time-to-market and shorten release cycles in their product roadmap. Based on this rationale, it is expected that first 5GC solution suites will be quickly upgraded with new features, including multi-slice support in AMF/SMF. This feature allows the configuration of two or more slices in the same 5GC, by fetching associated S-NSSAIs from the NSSF and injecting them into the corresponding AMF/SMF instances. The ability of having multiple 5GC slices (CN slice subnets) running in parallel may offer operators greater possibilities to tap new 5G use cases targeting public network users (B2C market) and industry companies (B2B market). These customers may have different service requirements in terms of performance and functionality, hence the need to define different 5GC slice types for them:

1. Business-to-Customer (B2C) slice types, used for serving traffic from user-centric applications.
2. Business-to-Business (B2B) slice types, used for the provisioning of non-public networks (NPNs) [44], in particular for public network integrated NPNs (PNI-NPNs) [45].

**Figure 11:**
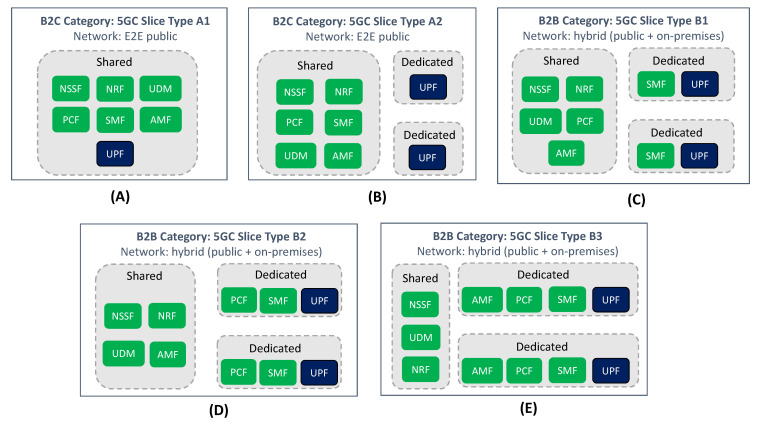
5GC slice types. (**A**,**B**) represents B2C slice types. (**C**–**E**) represents B2B slice types.

Figure 11 captures a representative number of these 5GC slices types.

On the one hand, there is the *B2C category* (Figure 11A,B). This category includes 5GC slices (i) designed for end user consumption, so there is no need to have in-slice dedicated control plane functions; and (ii) to be entirely built on the 5G public network infrastructure (PLMN). Within this category, two different slice types can be found.

- **5GC slice Type A1 (shared UPF)**: deployment flavor wherein the 5GC slice does not have a dedicated UPF; indeed, the in-slice UPF instance is also shared with other 5GC slices.
- **5GC slice Type A2 (dedicated UPF)**: deployment flavor wherein the 5GC slice is allocated with a separate UPF.

In the B2C category, the first 5GC slices to be launched may be of Type A2, with few slices hosting premium communication services that end users can subscribe to. With a commercial model based on offering VIP service experiences, the operator looks to keep existing users and attract new ones, generating moderate revenues from their subscriptions in the short term.

For Type A1 slices, the situation is rather different. Unlike Type A2 slices, where traffic isolation across them is preserved with the provision of dedicated UPFs, in Type A1 slices the UPF is shared among them. In this context, operators expect that one UPF can support multiple 5GC slices, which means that one UPF shall be able to manage user plane resources (e.g., UE IP addresses, GTP-U Fully Qualified TEIDs, CPU, memory, bandwidth, etc.) at the S-NSSAI granularity. Unfortunately, the ability for a UPF to perform resource management (e.g., resource separation, resource allocation, resource usage report) per 5GC slice is not yet available in the standards due to technology limitations inherent to UPF internals, though 3GPP have already started working on solutions to solve this [46]. Being a Rel-17+ feature, we are still years away from seeing Type A1 slices running in production networks. However, their availability will mark a major turning point in operator B2C slicing strategies, with the ability to deploy 5GC slices at a much wider scale, offering performance levels similar to Type A2 slices, but using a much lower number of UPFs. Additionally, the operator can use Type A1 slices to aggregate users with similar performance profiles, all this in a transparent manner, in search of creating efficiency improvements. This use of slicing, which allows the operator to streamline network management operations, is referred to as ’Network Slicing for Network Operator internals’ in [17].

On the other hand, there is the *B2B category* (Figure 11C–E), which covers all the 5GC slices which are intended for industry customers. Examples of these customers include verticals and hyperscalers. Unlike the B2C category, the 5GC slices belonging to this new category will be used to host services for private use (i.e., only available for the customer’s subscribers), which typically span beyond the operator’s service footprint. This means that (i) every slice shall have dedicated control plane functions, so that the isolation of traffic management can be preserved across slices, and (ii) not all the in-slice functions will be hosted by PLMN; indeed, some of them might be deployed at customer facilities (e.g., UPF). Within this category, three different 5GC slice types are worth mentioning.

- **5GC slice Type B1 (baseline CP)**: deployment flavor wherein 5GC slice is provided with dedicated UPF and a dedicated SMF. This ensures that in-slice traffic flows have an independent management and configuration, completely separated from other 5GC slices.
- **5GC slice Type B2 (advanced CP)**: represents a 5GC slice Type B1 provisioned with dedicated PCF. Having a slice-specific PCF allows the customer to inject tailored QoS policies over in-slice traffic flows.
- **5GC slice Type B3 (premium CP)**: represents a 5GC slice Type B2 provisioned with dedicated AMF. Having a slice-specific AMF allows the customer to retain full control over mobility and connection management aspects regarding their subscribers.

The B2B category aims to exploit the real benefits that network slicing enables, which is the ability to provide separate network partitions with independent management for different industry customers. What these customers value most is to perceive allocated 5GC slices as dedicated, self-contained networks, under their own control. To that end, it is important for the operators to ensure that 5GC slices are delivered with network capabilities equivalent to those offered by private 5GC solutions (e.g., guaranteed SLA, traffic separation, controllable and configurable network), but at a much more reduced cost. This, together with the trust on operator’s proven know-how on OAM activities, is what will drive B2B customers to ask for a 5GC slice rather than purchasing a private 5GC from a 3rd party.

As evidenced from Figure 11C–E, the customer’s perception of having a dedicated network requires the operator to provision 5GC slices with separate instances of some network functions. With the cloud-native design of 5GC and the consolidation of NFV practices into container-based environments, operators are conducting trials in this direction, assessing how the allocation of dedicated network functions impacts the number of 5GC slices that can be instantiated. This isolation vs scalability trade-off has demonstrated that the most optimal solution is to provision 5GC slices with dedicated instances of UPF and SMF. This flavor, which corresponds to 5GC slice type B1 (Figure 11C), will satisfy the service requirements of most industry customers [47,48]. However, there also exist specific customers whose business requirements may make them ask operators for more tailored 5GC slices, such as type B2 slices (Figure 11D) and type B3 slices (Figure 11E). Examples of these business requirements include the need for the customer to keep full control of QoS policies, or the need to get separate connection management of their subscribers.

Putting the B2B and B2C category solutions into the timeline reflected in Figure 9, we can outline two things. First, in relation to the B2C category, we can see that type A2 slices may be commercially ready in the short term, while type A1 slices are expected in the long run. Secondly, in relation to the B2B category, we can see that all 5GC slice types may be available in the medium term, once Rel-16 features are integrated into the 5GC. Unlike the B2C category, where the difference between 5GC slice types is subjected to the availability of 3GPP solutions, in the B2B category the technology maturity of all the 5GC slice types is the same. The decision of going for one or another solution is entirely dependent on the customer-specific business requirements.

### 5.2. Add-on Features

The 5GC arena provides a lot of value-added capabilities that have a direct impact on the use of slicing, and that operators can progressively incorporate into their commercial networks.

In the short term (i.e., deploy ring in the radar), operators are focused on the introduction of edge computing. Edge computing is an evolution of cloud computing that allows moving workloads from centralized data centers (e.g., central PoPs) down to the telco edge nodes (e.g., regional PoPs), closer to consumers. In network slicing, edge computing is a must; in fact, bringing application hosting closer to the UE’s access point of attachment allows achieving an efficient service delivery through the reduced end-to-end latency and load on the TN. The capabilities enabled by edge computing technology can be clustered into two solution sets:

- **Baseline edge computing**: provides support for application hosting and user-to-application connectivity. To that end, two network capabilities are needed. On the one hand, application placement capability, which allows for the optimized deployment of service applications at the target edge node, based on criteria such as resource availability, geographical areas, cost and latency requirements. On the other hand, edge node discovery capability, which represents the ability to identify an edge node capable of serving application clients (running on devices). In fact, when an application client wants to connect to an application, there is a need to discover the optimal edge node, which is the one that runs instances of the application, has the necessary resources (CPU, GPU, etc.) and provides the lowest network latency. For this discovery, there exists two solutions: DNS based (network layer solution, specified by 3GPP SA2) and device based (application layer solution, specified by 3GPP SA6). For further information on the pros and cons of these solutions, see [49].
- **Advanced edge computing**: provides mobility support in edge computing scenarios. As the user moves, it might happen that the current edge node is no longer valid, either because of SLA violation (e.g., the latency between the UE and serving node exceeds the maximum delay budget) or maintenance reasons (e.g., a node failure). This situation results in the user moving to a new edge node, a process that needs to be completed with the premise of keeping a seamless user service experience. This requires the availability of three main network capabilities: (i) service continuity capability; (ii) application re-location capability, i.e., to move the VM/container hosting the application instance from the source to the target edge node; (iii) context migration capability, i.e., to transfer the context from the stateful application towards the target edge node.

In the medium term (i.e., test ring in the radar), as long as standards and commercial products mature, operators are expected to enrich slicing functionality with the following solutions:

- **NWDAF**: the Network Data Analytics Function (NWDAF) [50] is a 3GPP Rel-16 function that provides network analysis information (upon request) about 5GC network entities. It provides S-NSSAI level analytics, and hence it may become the entry point to realize Artificial Intelligence (AI) in 5GC slices. NWDAF consumers can query for slice load levels and slice QoE measurements, or subscribe to slice-specific notifications that provide periodic updates or anomaly alerts. As shown in Figure 4, examples of NWDAF consumers include: the Network Slice Selection Function (NSSF), which uses the S-NSSAI level analytics to add real-time intelligence to its slice selection algorithms; the PCF, which makes use of NWDAF info to optimize policy decisions on individual 5GC slices; and the NSSMF. 3GPP TS 23.288 [51] reports use cases on the use of NWDAF to extract network analytics on a per network slice level.
- **Multiple slices per UE**: though 3GPP Rel-15 specifications allow a device to connect up to eight slices at the same time, thanks to the introduction of URSP (see Section 3.1) the reality is that most Rel-15 commercial solutions do not allow this feature. The existing limitations in commercial 5G SA handheld terminals prevent an UE from being connected to more than one slice at the same time. These limitations bet on the device’s Operation System (OS) [29]. The device’s OS mediates between the application clients and the device’s modem, where the URSP is installed. Operators, vendors, device manufacturers and chipset providers are working together to find workarounds, with de-facto solutions currently being assessed in different PoCs. Among these solutions is the 5G slicing support in Android 12(S) devices, announced by Google in October 2021. (https://cloud.google.com/blog/topics/telecommunications/5g-network-slicing-with-google-android-enterprise-and-cloud, accessed on 20 October 2021).
- **Secondary authentication**: Network Slice Specific Authentication and Authorization (NSSAA) attribute is defined in the GST [21] to specify whether, for a network slice, registered devices need to be authenticated by an external AAA server (Authentication, Authorization, Accounting server) using credentials different than the ones used for the primary authentication. This add-on feature, first introduced in 3GPP Rel-16 specifications, is intended for those industry customers that want to perform a second authentication over their subscribers. Operators are conducting bilateral trials with customers to help them understand the value of integrating NSSAA in B2B category 5GC slices, especially when used in the context of PNI-NPNs. In these trials, the operator-owned 5GC’s NSSAA Function [52] contacts with the customer-owned AAA server via an AAA proxy. For further details on this interaction, see [10,30].

Finally, in the longer run (i.e., explore ring in the radar), the integration of Rel-17+ features into the 5GC will allow operators to unleash the full potential of 5GC slicing. In this scouting phase, operators are have set their sights on these two featured solutions.

- **Slice Roaming**: operators are expected to support roaming for network slicing, at least for network slices deployed from S-NESTs. However, this feature is still years away from being in commercial networks, as there are technical and commercial aspects that need to be agreed upon. The technical aspects are discussed in [53], a GSMA document where operators have captured their priorities on slice roaming so as to guide the specification of normative solutions in 3GPP. The commercial aspects include charging, billing and business models that are still under discussion. Unless all these aspects are agreed and reported, no multi-operator trials are expected shortly.
- **NSACF**: the Network Slice Access Control Function (NSACF) is a Rel-17 5GC function that monitors and controls (i) the number of registred UEs per network slice, and (ii) the number of PDU sessions per network slice [10]. With the NSACF, operators can enforce quotas on individual slices, making sure the signalling traffic and packet flows do not exceed the maximum slice load. NSCAF is still in stage 2 (functional definition), so no vendor solutions are yet available. In the mean time, operators are now trying to understand how to best apply this functionality to improve internal network operation, and how to link them with the admission control functionality at the NG-RAN side.

## 6. RAN Domain

In the RAN domain, the technology radar puts the focus on three different dimensions: functionality, radio resource allocation and penetration. The *functionality* dimension provides a deep dive on the applicability of open RAN principles on NR protocol stack functions to design and configure RAN slices, going from monolithic solutions towards more flexible, service-tailored composition patterns. The *radio resource allocation* dimension discusses the availability of solutions to segregate and dispatch cell radio resources to competing RAN slices, so that their targeted KPIs are met. Finally, the *penetration* dimension refers to the penetration of RAN slicing technology within the operator’s footprint.

### 6.1. Functionality

As noted from Figure 2, the target RAN slicing architecture lies on the possibility of (i) having a 3-tier NR protocol stack, distributed into RU, DU and CU modules; and (ii) provisioning a dedicated CU-UP instance to each slice, with tailored PDCP configuration settings, so that the delay and security requirements for a given S-NSSAI can be fulfilled. The achievement of these two milestones is mandatory for an operator to have a fully operable RAN slicing solution, as described later on. For the sake of network efficiency (i.e., OPEX savings) or further service innovation (i.e., new revenue streams), the operator might decide to enhance the baseline solution by integrating add-on features atop. One example is the integration of the RAN Intelligent Controller (RIC) [54], an optional AI-powered functionality originally defined in the O-RAN framework [55].

In the following, we describe the stepwise journey we foresee for a future-proof RAN slicing.

In current NSA scenarios (i.e., as-is ring in the radar), the predominant operator scenario is a few **physical gNBs** providing macro coverage to city and suburban areas. Installed in strategic geographic locations, these gNBs have inbuilt 4G/5G essential features, including massive MIMO, Dynamic Spectrum Sharing (DSS) [56], Narrow Band IoT (NB-IoT) and RAN sharing.

As soon as the 5G coverage footprint needs to be extended, something that has already started with the rollout of first commercial SA networks, operators may migrate towards gNB cloudification, in search of CAPEX reduction. In fact, with this action, the operators are able to extend 5G coverage at large scale without the need to deploy costly physical gNBs everywhere. This bets on different, yet intertwined solutions:

- **DU-CU disaggregation**, whereby gNB is functionally split into one (centralized) CU instance and multiple (distributed) DU instances, conforming to a split 2 option. The result of this disaggregation is that CU can be entirely implemented in software, and therefore deployed as a VNF in any cloud environment. In fact, while individual DU instances remain colocated with RU at cell sites, the workload corresponding to the CU instance can be moved to the telco edge cloud.
- **Control User Plane Separation**, whereby the virtualized CU software is further decomposed into one CU-CP instance and multiple CU-UP instances. This requires a complete reshaping of CU software design, transforming a coarse-grained (VM-based) VNF into a number of modular (container-based) VNFs, each hosting a different instance.

These two solutions will be available in the short term, hence they are categorized in the deploy ring of the radar.

In the medium term, once the penetration rate of cloudified gNBs is significant, the operators may use deployed assets to have a fully operable RAN slicing environment. This environment, which shall be compliant with the two requirements captured in the beginning of this subsection, leverage on two solutions:

- **Slice-specific vCU-UP**, based on providing each RAN slice with a separate vCU-UP instance. This solution not only ensures user plane traffic isolation across different RAN slices [57], but also adapts/customizes the processing of DRB flows according to the slice specific needs.
- The implementation **vDUs**, to allow for a 3-tier NR protocol stack. This solution is the result of a three-step journey, whereby the DU is first separated from the RU (introducing a fronthaul link between them, according to split option 7-2x), then designed in software (modelled as a VNF), and finally deployed into access PoPs (far edge sites). The ability to move DU workloads into a cloud environment is of particular interest for large-scale slices hosting distributed eMBB services or mIoT applications. However, this feature does not come like that alone; indeed, it needs to be accompanied with the provision of rich-featured CPU architectures and hardware acceleration solutions [27] in the access PoP, as outlined in Section 2.4. These assets are aimed at reducing the impact that virtualization overheads may introduce on DU packet-processing performance.

**Figure 12:**
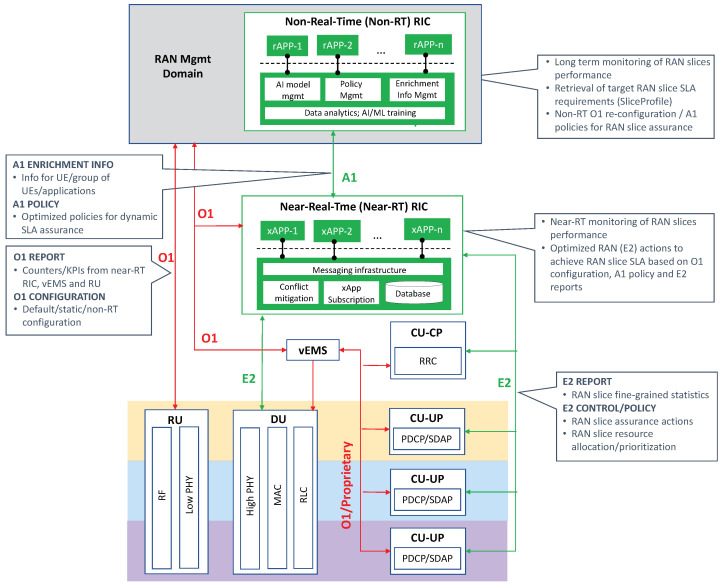
AI assisted RAN slice resource control.

Industry stakeholders (including operators) have started to test these two solutions, validating their performance and assessing their techno-economic viability in typical scenarios where the vDU is shared across slices. In parallel, operators have launched a new activity on RIC, pushed by the O-RAN momentum. Unlike the first workstream, focused on searching for a baseline RAN slicing environment that can be easily scaled and replicated across the operator’s service footprint, this new workstream has the goal of checking how the RIC can further enhance RAN slicing features. As noted in [55], the RIC is a non-mandatory O-RAN component consisting of two modules: (i) near-RT RIC, which hosts ms-level RRM logic with embedded intelligence; and (ii) non-RT RIC, which provides delay-tolerant functionality including service and policy management, RAN analytics and AI/ML model training. Figure 12 provides details on these two modules. In this testing phase, the focus is on the **near-RT RIC** (near-Real Time RIC), and in particular on the capabilities of the E2 interface [58].

In the long run, when the commercial O-RAN solutions are relatively stable, operators may focus on how to include the entire RIC in the day-to-day operation of RAN slices, making it more agile and intelligent [59]. In this regard, the following solutions are currently being explored:

- **xApps**. As seen from Figure 12, the near-RT RIC delivers a robust, scalable and secure platform for xApp hosting. These xApps are third party control applications that complement traditional RRM functionality, by bringing advanced algorithms applicable to real-time use cases, including QoS/QoE optimization, per-UE controlled load balancing, traffic steering and seamless handover control. Operators together with third party developers are exploring the possibility of implementing slice-specific RRM procedures through xApps, in order to decouple life cycles of slicing related innovation (e.g., 4/6-month release cycle) from CU-CP hosted RRM policies (e.g., 1/2-year release cycle).
- **Non-RT RIC**. The non-RT RIC (non-Real Time RIC) will be the main driver for AI-powered RAN slicing. This module will host and manage all AI/ML models that will later be pushed into the near-RT RIC down to the individual RAN slices. In the exploratory phase, where the operators are now, the main entry barrier they have encountered is its complex integration. In fact, the non-RT RIC needs to communicate with (i) the RU and the vEMS, using O1 interface [60]; (ii) the near-RT RIC, using A1 reference point [61]. In addition, it needs to interact with 3GPP management system, something that today is still under discussion in O-RAN community.

Figure 12 illustrates the integration of all the O-RAN components into the network slicing system architecture, including the reference points (E2, O1 and A1 interfaces) across them. To illustrate the information exchanged between these interfaces, we propose an example of the use of the RIC to make an AI assisted resource control for RAN slice SLA assurance. The logic of this resource control is hosted by xApps. Further details on this example can be found at the end of Section 6.2, once the details on radio resource allocation are explained.

### 6.2. Radio Resource Allocation

In this dimension, we encounter a number of solutions in the gNB scheduler enabling RAN slicing. These solutions are based on two different yet complementary mechanisms: priority scheduling and radio resource partitioning.

On the one hand, *priority scheduling* is a mechanism used for service differentiation between RAN slices when they all compete for the same PRBs. This corresponds to best effort slices, as captured in Table 2. The service differentiation here is achieved with the definition of multiple DRB profiles, each featured with a different scheduling priority, and their allocation to existing RAN slices. This approach allows giving one or more RAN slices a preferential treatment with relative priority to others. The policy for DRB profile allocation is based on QoS criteria, though other business-wise criteria can be applied (e.g., based on platinum/gold/silver subscription).

On the other hand, *radio resource partitioning* is a mechanism based on PRB reservation. Unlike priority scheduling, radio resource partitioning allows segregating cell resources across different RAN slices. Here, resource segregation consists of allocating separate PRB chunks to the DRBs associated to these slices. For this PRB allocation, the gNB scheduler configures the resource quotas per slice. As shown in Figure 6, the operator can configure up to three resource quotas in a slice, namely, maximum slice quota (mandatory), minimum slice quota (mandatory), and dedicated minimum slice quota (optional). For the definition of these per slice quotas, the operator makes use of RRMPolicy class defined in the 5G NRM, by applying the RRMPolicyRatio attributes (i.e., RRMPolicyMaxRatio, RRMPolicyMinRatio, RRMPolicyDedicatedRadio) to the {S-NSSAI, PLMN ID} tuple the RAN slice is associated with. For further information on this mapping, see clause 4.3.36 from 3GPP TS 28.541 [15].

Table 3 provides a comparative analysis between priority scheduling and radio resource partitioning. While it may seem logical to define PRB reservation for each slice supported at the RAN, this is, in practice, suboptimal. There is a trade-off in performance between the gain from dedicating resources to specific slice services and the overhead in maintaining numerous resource partitions. The balance is to keep sufficient PRB chunks to guarantee resource isolation per RAN slice when needed, while not impacting radio performance due to excessive partitioning.

In the following, we describe the solutions that we foresee from these two RAN slicing mechanisms, and that are illustrated in the radar shown in Figure 9.

In today’s dominant NSA scenarios (as-is ring in the radar), the only possible solution for RAN slicing is **QCI Priority Scheduling**, based on giving a preferential treatment to those DRBs associated with top-priority services. The priority of a service depends on the QoS parameters associated with the service flows, with a QCI as key parameter, complemented with other QoS related info (i.e., GBR + MBR values if service flows convey GBR traffic, AMBR values if service flows convey non-GBR traffic). Upon computation on the EPC side, the priorities of different services are sent to the gNB, which uses them to define corresponding DRB profiles. The number of DRB profiles available in current production networks is rather low; the reason is a coarse-grained design of DRBs, and that only few of them convey prioritized traffic.

**Figure 13:**
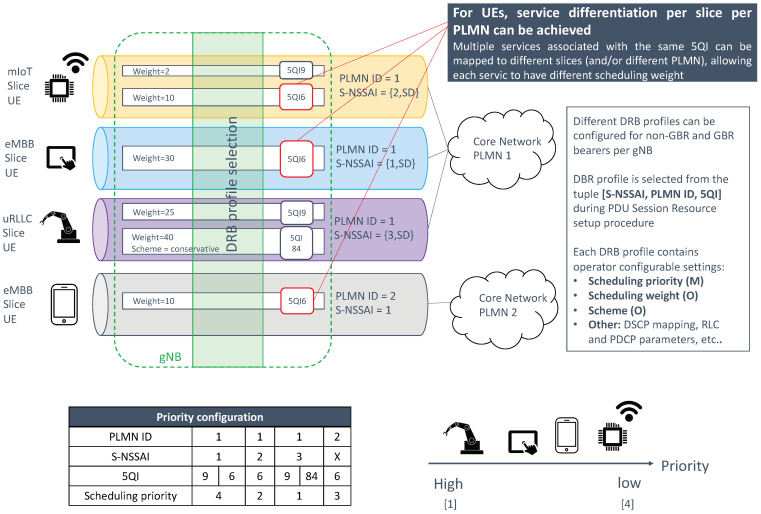
Priority Scheduling.

As we move towards the rollout of the first 5G SA networks, new priority scheduling solutions are appearing:

- **5QI Priority Scheduling**. This solution is equivalent to the QCI Priority Scheduling, but using 5GC, which provides much more granular QoS control than the EPC. The fact that (i) the 3GPP 5G QoS framework allows one slice to convey multiple QoS flows, each featured with a specific 5QI, and (ii) the DRB profiles are computed based on 5QI, make it possible to have intra-slice service differentiation. Figure 13 illustrates an example of how this solution works.
- **Relative Priority Scheduling**, which is an evolution of the previous solution. This evolution is based on enriching DRB profile characterization with additional configurable settings, including scheduling weight and scheme, among others. These settings, listed in Figure 13 with the optional “(O)” tag, allow the gNB scheduler to resolve conflicting situations that are beyond the capabilities of 5QI priority scheduling. One example is when QoS flows from different slices have the same 5QI, but there is a need for preferential treatment of one of these slices. In such a case, scheduling weight can be used, as elaborated in the top-right corner of Figure 13.

**Figure 14:**
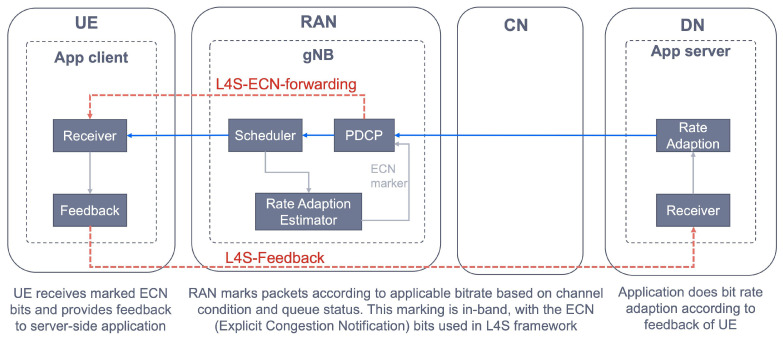
Feedback loop for delay controlled priority scheduling, with in-band L4S.

These two solutions build up the RAN slicing suite that operators have started to deploy, and may be operationally ready in the short term. However, with sights already set on evolving slicing features in the medium term, operators are also testing other solutions:

- **Delay controlled Priority Scheduling**. This solution consists of enriching gNB scheduling logic with the adaptive managed latency concept. It allows providing better experience to Rel-16 services, mostly interactive services that require high data rate and low latency communications. The adaptive managed latency concept represents the ability to provide bounded and steady low latency for these services, by coupling gNB scheduler and application in a feedback loop with dynamic rate adaptation signalled by the service application. This coupling can be enforced using either vendor-specific mechanisms or standard frameworks, such as Low Latency, Low Loss, or Scalable Throughput (L4S) [62]. Figure 14 depicts how L4S congestion marking and feedback can be applied for gNB scheduling. Note that this Delay Controlled Priority Scheduling solution (application layer rate adaptation) is complementary to Relative Priority Scheduling (network layer service differentiation), and both can be used simultaneously.
- **Hard Radio Resource Partitioning**. This is the first solution from the radio resource partitioning category. For the PRB allocation, this solution assumes that individual slices are only configured with dedicated resources, without prioritized resources. To achieve this, the operator sets the same value for the dedicated minimum slice quota (RRMPolicyDedicatedRatio) and the minimum slice quota (RRMPolicyMinRatio). From the set of flavours tabulated in Table 2, one can note that the RAN slices resulting from this solution are compliant with the “dedicated slice-profile 1” settings. This flavor provides isolation and secure resources in high load conditions, at the cost of poor multiplexing gains. The reason is that dedicated resources of a slice cannot be used by others, even though the slice resource usage is below the dedicated minimum slice quota.

Finally, in the long run, we can find fully-blown RAN slicing solutions. These solutions, listed below, are years away from being commercially available; in fact, they are still taking shape in the reference standards: 3GPP and O-RAN Alliance. Therefore, they are captured in the explore ring in the radar.

**Figure 15:**
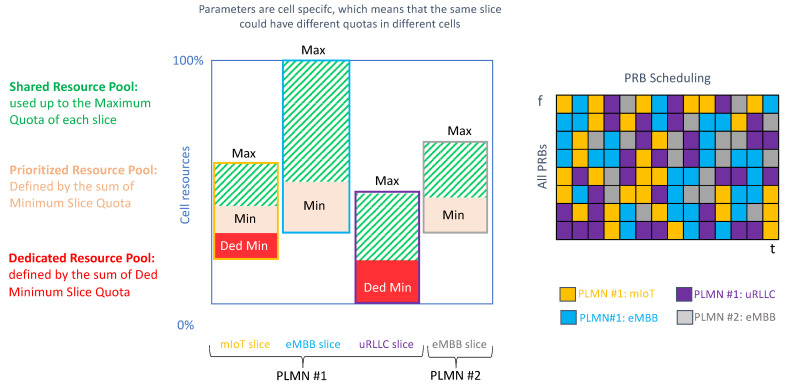
Radio Resource Partitioning.

- **Static (Policy-based) Flexible Radio Resource Partitioning**. It allows for defining prioritized resources per slice, a feature which was disabled in the Hard Radio Resource Partitioning. With this new option, RAN slices can now be configured according to “dedicated slice-profile 2” and “prioritized slice” settings, as shown in Table 2. The definition of prioritized resources per slice boosts resource efficiency, at the cost of making gNB scheduler logic much more complex, with a larger number of decision-making variables that need to be computed in real-time.
  It is important to note that gNB scheduler can work with both hard and flexible radio resource partitioning solutions, as depicted in Figure 15. This example shows that the gNB is configured to serve four slices from two different PLMNs: PLMN#1, hosting mIoT, eMBB and uRLLC slices, and PLMN#2, hosting another eMBB slice. Looking at the slice specific quotas, one can note that the uRLLC slice is scheduled with hard approaches. For the remaining slices, flexible resource radio resource partitioning is applied, with the mIoT slice configured with “dedicated slice-profile 2” flavor and the eMBB ones configured with “prioritized slice” flavors.
- **Dynamic (AI-assisted) Flexible Radio Resource Partitioning**. This solution takes the previous solution to the next level, with the possibility of changing slice resource quotas over time, depending on collected performance metrics. To cope with this dynamism, operators need to take humans out of the loop, replacing them with novel AI-assisted artifacts enabling closed-loop automation. The xApps hosted by the near-RT RIC are perfect candidates for this role; indeed, they can provide agility and context-awareness in the decisions of changing resource quotas [63].

Figure 12 illustrates an example of the usability of near-RT RIC for real-time resource control of operative RAN slices. As seen, the near-RT-RIC makes use of E2 interface to interact with associated DU, CU-UP and CU-CP instances, for both monitoring (E2 REPORT) and configuration management (E2 CONTROL/POLICY) actions. The governance of these actions lies on xApps. The logic of these xApps can be assisted with AI/ML models, which are made available for consumption by the non-RT RIC using the A1 interface. By comparing collected metrics against AI/ML models, the xApps can make real-time decisions on quota changes, enforcing them in corresponding CU-UP instances. Though the ultimate invocation and execution of AI/ML models lies on xApps, this cannot be done without the Non-RT RIC, which plays a key role in this workflow; indeed, non-RT RIC is responsible for the management of individual AI/ML model (e.g., AI/ML model deployment, configuration, performance evaluation, termination, validation and testing).

### 6.3. Penetration

In this section, we provide a description of the penetration path we foresee for network slicing in cell sites. For this exercise, it is important to take into account the environment setup. In this regard, we consider three phases: early introduction (phase 1), full adoption on standalone private 5G networks (phase 2) and full adoption in public 5G networks (phase 3).

The *phase 1* covers the as-is and deploy rings of the radar. In this phase, it is assumed a baseline RAN slicing solution, consisting of applying 5QI priority scheduling (see radio resource allocation dimension) over cloud NG-RAN scenarios (see functionality dimensions), with no mobility support. This setup has minimal impact on production networks, without compromising backwards compatibility, and therefore constitutes a good candidate for early introduction in the PLMN. The integration of baseline RAN slicing in first SA networks ensures QoS-based service differentiation on RAN side, which is essential for operators to start commercializing 5GC Type A2 slices for B2C market (see Figure 11).

However, for more elaborated setups leveraging open RAN principles, the above rationale is no longer valid. The major changes that these setups bring in RAN planning and configuration patterns prevent their large-scale introduction in today’s commercial networks, which account for a large percentage of legacy RAN assets. Instead, operators may initially choose to start testing them RAN slicing on private networks (phase 2) before moving to macro cells (phase 3).

The *phase 2* covers the deploy, test and explore rings of the radar. In this phase, the operator may integrate RAN slicing solutions only in greenfield environments, **on-premises**, creating isolated islands of private 5G adoption. This setup constitutes a perfect niche for the operators to start commercializing their innovative RAN slicing solutions towards B2B customers, as the radio resource allocation mechanisms and O-RAN components become available. These solutions can be used either (i) to provide traffic isolation across on-premises customer services, or (ii) to provide the customer with a guaranteed SLA between the device and 5GC. In the second case, RAN slicing is used in conjunction with the B2B category 5GC slices to provide PNI-NPN services.

Finally, there is the *phase 3*, spanning across the test and explore rings of the radar. This phase 3 consists of applying the lessons learnt in phase 2 to macro cells. In fact, the trials in phase 2 will serve the operator as a playground before moving to the PLMN, where UE density and traffic patterns are radically different, and where mobility events now need to be taken into account. To facilitate this transition, a three-step journey is foreseen:

- **PLMN, single-slice TAI**. The operators will start to replicate the trials in specific tracking areas, with their cells configured with one single S-NSSAI.
- **PLMN, multiple-slice TAI**. The same solution as the previous one, but configuring all the cells from the same tracking area with two or more S-NSSAIs.
- **PLMN, large-scale**. The lessons learnt from the small-scale deployments allow improving RAN slicing before massive adoption in the PLMN, where more complex problems on capacity planning and mobility management may appear.

## 7. TN Domain

In the TN domain, the technology radar requires the analysis from two separate dimensions: transport technologies and transport SDN fabric. The *transport technologies* dimension captures the protocol encapsulation and data plane solutions across the different network segments. On the other hand, the *transport SDN fabric* dimension refers to the control and management plane aspects, discussing the application of programmability and automation through SDN.

These two dimensions are not coupled but complementary, in the sense they can evolve at a different pace and they do not necessarily need to be used together. The transport technology dimension includes all the Layer 1/2/3 solutions that allow segregating connectivity resources and enforcing traffic separation at the WAN infrastructure, therefore enabling TN slicing realization. The sole use of these transport technologies is enough to have a sliced WAN infrastructure, but not to get it provisioned and operated in a dynamic way. The latter would require implementing programmability and automation capabilities atop these technologies, something that can only be brought by the SDN paradigm. The complementarity of the transport SDN fabric lies in the ability to operate with these technologies using a set of SDN controllers instead of traditional, siloed TN management systems (which typically lead to rather static, manual configurations). This requires equipping these SDN controllers with programmatic interfaces on the southbound side, towards the underlying technology devices. These interfaces shall be developed in such a manner that SDN controllers can inject configuration actions and retrieve collected data following a model-based approach.

### 7.1. Transport Technologies

The system architecture represented in Figure 2 shows the entities on the path for an E2E slice, assuming a 3-tier deployment for the gNB: RU, DU and CU-UP. As seen, the user plane from the device to the application includes the following TN segments: fronthaul (O-RAN fronthaul interface), midhaul (3GPP F1-U interface), backhaul (3GPP N3 interface) and DN (3GPP N6 interface). The F1-U and N3 interfaces use GTP-U to transport packets (IPv4, IPv6, Ethernet or unstructured) in the PDU session established between the UE and the DN. The fronthaul interface carries the radio frames in the form of In-Phase (I) and Quadrature (Q) samples, using eCPRI [64] encapsulation over Ethernet or UDP over IP.

The mission of TN slicing is to implement slicing-featured capabilities across all these connectivity segments, ensuring that the SLA requirements are met not only at the 3GPP network function layer, but also at the transport underlay. Examples of these capabilities include guaranteed QoS (e.g., bandwidth reservation, upper latency bounds, controller delay variation), isolation, protection and reliability (e.g., disjoint routes).

As outlined in Section 2.3 and shown in Figure 8, there exist multiple networking solutions that can be used for the provision of these capabilities, ranging from soft slicing to hard slicing, with some trade-off solutions in between (i.e., hybrid slicing). Next, we present the journey we foresee towards the realization of an end-to-end TN slicing, based on a step-wise integration of different transport technologies into carrier networks.

Today’s scenarios are based on 5G NSA, where the slice data path only includes two segments: backhaul segment (between the physical gNB and S/P-GW) and DN segment (between S/P-GW and application). Typical implementations for these scenarios bet on the use of **traditional L2/L3 overlay** solutions, mainly VPN techniques. Layer 2 VPN techniques are, for example, Virtual Private LAN Service (VPLS), which sets up a Ethernet-based communication over MPLS tunnels, or Virtual Leased Line (VLL), which emulates a pipeline between two given endpoints. Layer 3 VPNs can be realized, for example, via Virtual Private Routed Networks (VPRN), featuring MPLS-based VPNs. Two main reasons explain the preference for these technologies. On the one hand, they do not have an impact in the underlay. Taking into account the brownfield design of today’s transport networks, it is natural for operators to reuse as much as possible existing overlay technologies, especially if they are carrier-grade and widely available along the operator footprint. On the other hand, they are cost-efficient, with a performance that is enough to satisfy the transport requirements of NSA slices.

In the short term, early B2C slicing offering will require evolving transport networks with different solutions such as the listed below.

- **DiffServ Code Point (DSCP)**. The integration of 5GC will lead to a change in the backhaul segment, now based in the N3 interface. Additionally, the DU-CU disaggregation will produce the F1 interface for the mid-haul segment. With this scenario, the setup is as follows: a GTP tunnel encapsulating user packets with slicing information captured in the {PLMN ID, S-NSSAI, 5QI} triplet transverses the backhaul and midhaul segments. Since the IP underlay across these segments is not able to interpret these 3GPP signalling identifiers, it is not possible for the border router (see Figure 7A) to apply the constraints represented by this tuple. In this regard, the UPF and RAN shall perform transport level packet marking in downlink and uplink, respectively, by setting the DSCP in the outer IP header [65]. This information is used by the corresponding border routers in the IP underlay to differentiate traffic from different slices [66,67].
- **Segment Routing (SR)**, and its use across backhaul and midhaul segments. Apart from the rich built-in features (e.g., highly scalable, responsive, programmability) [68], what makes SR [69] an ideal technology option for the IP underlay is that it can be used with both the MPLS forwarding plane (SR-MPLS) and the IPv6 forwarding plane (SRv6). Examples of realization of slicing in SR networks can be found in [70,71]. The basis of this realization lies in the ability of SR to encapsulate additional information for discriminating traffic associated with different slices [72].

The solutions explained so far, belonging to the as-is and deploy rings in the technology radar, are part of the soft slicing category presented in Section 2.

In the medium term (the testing ring in the radar), as novel B2B offerings come into service portfolio and the slicing requirements get burdened, the capabilities offered by soft slicing solutions are not enough. In this regard, operators have started to evaluate new technologies, working on three main directions:

- **Hard slicing**. In this workstream, the operators are focused on trialing technologies like Flexible Ethernet (Flex-E), Flex-O and DWDM. These technologies are used to realize the concept of isolated traffic flows operating on common links that avoid negatively influencing the performance of each other in case of congestion. In particular, **Flex-E** [35] bets on the principle of calendar-based channelization, which consists of bundling or dividing physical Ethernet interfaces into multiple Ethernet hard pipes based on timeslot scheduling. The work in [73] gives further details on how Flex-E technology might be used to implement hard slicing. **Flex-O**, described in the ITU-T G.709.1/Y.1331.1 recommendation [74], provides Optical Transport Network (OTN) interfaces with comparable functionality to that of Flex-E based Ethernet interfaces. Finally, **DWDM** can be used for physical resource separation at wavelength level. Adaptive transponders over Wavelength Division Multiplexing (WDM), spectrum fragmentation and Optical Cross-Connect (OXC), and Reconfigurable Optical Add-Drop Multiplexer (ROADM) are optical network virtualization techniques that can be exploited for network slicing, so that wavelengths can be right-sized for the specific requirements of every slice.
- **Deterministic techniques**. Flex-O, Flex-E and DWDM are ideal for providing guarantees for throughput and delay, which will be a common pattern across most of the uRLLC scenarios. Apart from these hard slicing technologies, in order to enable deterministic real-time performance in transport networks, novel full-stack approaches are also proposed in the underlay, with techniques such as those outlined by the IEEE TSN Project Group (layer 2 aspects) and the IETF DetNet Working Group (layer 3 aspects). On the one hand, **TSN** is a set of IEEE 802.1 amendments that enable determinism of time-critical traffic flows, even in cases where traffic flows with different statistical characteristics are multiplexed. The use of slicing in TSN-based networks is discussed in [75]. On the other hand, **DetNet** defines a set of techniques to extend deterministic behavior to in-slice layer 3 paths. These techniques span from explicit routes, packet replication and elimination, to congestion protection with E2E synchronization [76].
- **SD-WAN**. It is not uncommon that B2B customers contracting 5GC slices will request QoS-assured connectivity across their enterprise sites. This setup may result in a multi-site B2B slice, typical in PNI-NPN scenarios. In this case, Software Defined WAN (SD-WAN) [77] is a good candidate solution for the DN segment. Some operators have started to execute PoCs in this direction, e.g., [78].

**Figure 16:**
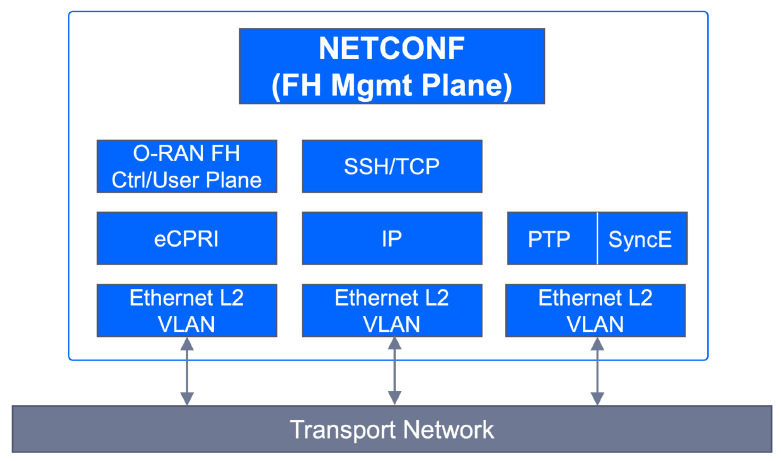
O-RAN FH interface protocol structure.

Finally, the focus in the longer term is on making **O-RAN fronthaul interface slicing-aware** [59]. Unlike the midhaul and backhaul segment (3GPP interfaces), where slicing information is injected with the the EP_Transport IOC (see Figure 7B), the fronthaul segment (O-RAN interface) does not currently support slicing features. Figure 16 shows the current protocol structure for the O-RAN fronthaul interface. As seen, the stack allows DU and RU to exchange (i) signalling information; (ii) user plane information, based on frequency-domain IQ samples; (iii) timing and synchronization information; and (iv) management plane information. However, no slicing related data is yet supported. The challenge that industry shall solve is how to encapsulate S-NSSAI (3GPP signalling information) in the O-RAN fronthaul interface, considering that this interface is out of the 3GPP specifications. O-RAN Working Group 4 is working towards this direction, though normative solutions (and therefore commercial solutions) are still far away.

### 7.2. Transport SDN Fabric

The use of a transport SDN fabric brings automatic network control and programmatic capabilities. These features are key for the operators in their need to cope with the agility and management complexity of slicing in their transport networks, with multiple vendor and technology solutions underneath.

As of today, the scope of SDN technology has been circumscribed inside the data center, using it to simplify the management of internal connectivity as the adoption of cloud solutions is consolidated. However, the applicability of SDN into transport networks is a completely radical approach, with the use of new protocols and different model-driven operational practices quite tied to the specificities of the WAN technologies present in today’s carrier facilities. Making transport SDN a reality is not an easy task. In this regard, many efforts have been made over the past few years in this direction, with telco industry actors working out promising but misaligned solutions. Proof of this is the large number of architecture frameworks defined so far, e.g., [79,80,81].

**Figure 17:**
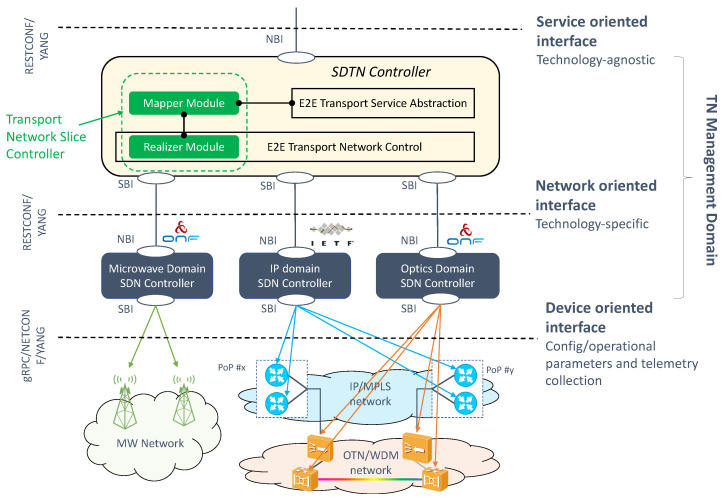
Transport SDN architecture.

It is now time to move forward and make progress. This requires the definition of a common strategy to reduce and select the most suitable standards to unify disparate SDN solutions into a single E2E, open transport SDN architecture. The preferred architecture option is shown in Figure 17. Originally proposed by Telefónica with the iFUSION brand [82], this architecture follows a hierarchical model, with specific domain controllers per technology domain (IP/MPLS, optical/DWDM and microwave/backhaul) and an overarching Software-Defined Transport Network controller (SDTN controller). This two-layer control architecture has become the reference framework for operators worldwide, as echoed in the white paper published by Telecom Infra Project’s Open Optical and Packet Transport (OOPT) project group [83].

The benefits of the targeted SDN architecture lie in the system design, built upon three principles.

- Technology domain controllers. The idea is to separate the control per technology concerns, then drive the different particularities of each technology with specific solutions suited to them. This separation of concerns also enables a higher scalability of the solution; in fact, in case a transport segment is divided among different administrative domains, multiple domain SDN controllers for the relevant transport segment might be included in the hierarchy, in a flexible way.
- Technology-agnostic SDTN controllers, abstracting the complexity below by offering a single entry point for programmability of the overall transport network. This entry point is accessed by the TN management domain’s consumers, which includes management functions from the rest of the management domains (see Figure 2).
- Seamless integration of network slicing features. These capabilities are to be provided by the Transport Network Slice Controller (T-NSC), which is an add-on component of the SDTN controller, as shown in Figure 17. This ensures that the SDTN controller can work with slicing and non-slicing services, by simply making use or bypassing the T-NSC, respectively.

In the following, we describe the journey towards the goal scenario: having the E2E, open transport SDN architecture depicted in Figure 17 up and running in commercial networks. We follow a bottom-approach for describing the approach taken.

In the lower part of the architecture, we see the device oriented interface, which corresponds to the SouthBound Interface (SBIs) of the individual domain SDN controllers. As with any programmatic interface, the SBI is composed of selecting a protocol to transfer the data, and YANG data models to define how the message is formed. In all the domain SDN controllers, the SBI uses NETCONF [84] protocol, the main protocol for device management operations. Further details are provided below:

- **IP domain SDN controller: SBI**. For the IP/MPLS segment, the solution is based on a single multi-vendor IP domain controller, charged with configuring the Layer-2 and Layer-3 network elements. The management of this equipment is done through the SBI, which bets on declarative configuration and model-driven operations using device YANG models, such as those those available in OpenConfig [85]. In addition to NETCONF/YANG, other protocols are considered in the SBI of an IP domain SDN controller, including (i) BGP-LS, to retrieve link-node topology of the IP/MPLS networks [86]; (ii) PCEP, to support Traffic Engineering [87]; and (iii) gRPC, to collect monitoring data [88].
- **Optics domain SDN controller: SBI**. For the optical segments, there is no way on having ’one-size-fits-all’ SDN controller. The reason is that transport DWDM networks are highly implementation dependent, with no practical interoperability at optical level. Having a programmatic NETCONF/YANG based SBI would require the disaggregation of existing optical transceivers and line-side components.
- **Microwave (MW) domain SDN controller: SBI**. Though the number of hops from end links to the fiber aggregation point is progressively shorted (especially with the new Integrated Access Backhaul solutions [89]), it is still common for operators to have multi-vendor aggregation paths in their MW underlay. The operation and configuration of these paths is rather manual and static, using the proprietary interfaces from vendor-specific network management systems. The goal of this workstream is to avoid the integration complexity and scalability burdens of this approach (i.e., with OSS needed to maintain multiple interfaces) by introducing a SDN controller, which is a vendor-agnostic configurator of the MW network. To that end, standard communication protocol and YANG device models are being considered on the SBI, turning it into a programmatic interface.

Going up in the architecture, we arrive to the network oriented interface, which permits the invocation of service fitting each technology underneath. This interface corresponds to the NorthBound Interface (NBIs) of the per-technology domain SDN controllers. The **Domain SDN controller: NBI** solution allows each controller to offer the following capabilities to the SDTN controller: (i) a vendor-agnostic provisioning interface, to request for the creation/deletion/modification of connectivity services; (ii) per-OSI layer topology and network inventory information; and (iii) active monitoring of network status, e.g., traffic statistics and event notifications. Unlike the SBIs, built with NETCONF operating over device YANG models, the NBIs bet on the use of RESTCONF [90] with network YANG models. On the one hand, the choice of RESTCONF (HTTP-based protocol) permits the reuse of all tooling around the REST interface, which is today the industry norm for non-device oriented configuration management operations. On the other hand, the network YANG models provide abstract representation of relationships between multiple devices, including topology and connectivity services. Table 4 captures the network YANG models that are for consideration at the domain controller NBIs.

Finally, consuming these NBIs, there is the SDTN controller. This controller orchestrates the domain specific capabilities to provide real-time control of multi-layer and multi-domain transport network resources. The efforts on this workstream go in three directions:

- **E2E SDN controller: internal logic**. The focus here is on the implementation of the E2E Transport Network Control block of the SDTN controller. The scope of this module can be summarized into five functionalities: (i) E2E control across the different domains, by coordinating the disparate technologies through their corresponding SDN domain controllers; (ii) per-layer E2E visualization, i.e., per-layer topology composition; (iii) stateful control of provisioned network services; (iv) multi-layer Path Computation Engine (PCE), which has the role of computing paths across multiple technologies based on the per-layer topology composition; and (v) service binding to transport resources, which enables the controller to obtain the best Traffic Engineering (TE) connections for a given transport connectivity service. An example of the functionality described in (v) is as follows: for a VPN having certain bandwidth and latency contraints, compute the set of Label Switched Paths (LSPs).
- **E2E SDN controller: NBI**. The focus here is on the E2E transport network abstraction block, in charge of exposing an abstracted topology view of the network resources and the available set of network services to SDTN consumers through an unified NBI. There is no solution yet in the standards, although quite close cooperation between Telecom Infra Project’s OOPT [83] and IETF’s TEAS [100] exists in this regard. Ongoing discussions reveal the idea to use LxSM models (see Table 4) as a starting point for the NBI implementation.
- **Slicing-aware E2E SDN controller**. This consists of incorporating the T-NSC, which will have the awareness of slicing at the transport layer. Though there is not yet an common view of what T-NSC represents, the telco industry agrees on the need (i) to align the T-NSC concept with the IETF Slice Controller developed by the IETF TEAS’s network slice design team [101], and (ii) to implement the T-NSC as an additional component of the SDTN controller. For the first point, the focus is to align the T-NSC with the use cases [102] and YANG network models [103] worked out for the IETF Slice Controller. For the second point, we foresee a solution similar to the one represented in Figure 17. As seen, the T-NSC might be built out of two separate modules: the mapper and the realizer. The mapper module is responsible for collecting the customer-facing view of the TN slice for further processing the TN slice request. Thus, this module would integrate the customer-facing view on the provider view for triggering configuration, control and management actions. On the other hand, there is the realizer module, which is in charge of coordinating different actions on a number of domain SDN controllers for effectively creating the TN slice, according to the original customer request. Integrated in the E2E Transport Network Control Abstraction block (see Figure 17), this module would manage the workflows for the TN slice provision, as well as for its life cycle.

Putting all the discussed solutions in the technology radar shown in Figure 9, we observe the following:

- The deploy ring includes **IP domain SDN controller: SBI** and **optics domain SDN controller: SBI** solutions. With the emergence of the first Rel-15 commercial networks, some operators have recently started the deployment of SDN technology in transport networks, although not at large scale yet. For now, it is limited to IP/MPLS and optical/DWDM segments, and highly coupled with specific use cases.
- The test ring covers **MW domain SDN controller: SBI**, **Domain SDN controller: NBI** and **E2E SDN controller: internal logic** solutions. The operators are currently conducting trials on features from these three solutions, which are expected to be available in the medium term.
- The explore ring captures the **E2E SDN controller: NBI** solution (for the integration of transport SDN fabric with the rest of OSS assets) and the **Slicing-aware E2E SDN controller** solution (to integrate the slice semantics in the transport SDN fabric).

According to the above rationale, it is clear that to get the targeted transport SDN architecture up and running in carrier networks, operators need to follow a staged approach where the full, E2E integration of slicing features constitutes the final stage. However, this does not mean we cannot have TN slicing meanwhile; indeed, as outlined in Section 7.1, there are multiple transport technologies available for slicing realization. What this really means is that the operator may not be able to have automation and programmability capabilities in the short and medium term when allocating and operating transport slices. The lack of SDN may force the operator to go for static provisioning and management operations, from the E2E management domain to technology-specific management systems, using traditional Command Line Interface (CLI) solutions with ad-hoc extensions to avoid vendor lock-in.

## 8. OSS Domain

Finally, for the OSS domain, the technology radar addresses two different dimensions: OAM and capability exposure. On the one hand, the *OAM* dimension refers to the set of activities related to network slice life cycle management. On the other hand, the *capability exposure* dimension touches on the need for providers to make network slice capabilities available for consumption to B2B customers, through easy-to-use service APIs.

### 8.1. OAM

The life cycle of a network slice is articulated into four different phases: preparation (slice design, slice on-boarding and network set-up), commissioning (slice instantiation and configuration), operation (slice activation, modification, reporting/supervision and de-activation) and decommissioning (slice termination) [17]. The operator’s main role is to manage the life cycle of all slices running in the network. In this endeavor, the operator makes use of OAM tools available in the OSS layer, as illustrated in Figure 2.

This section outlines the main solutions that enable the OSS to conduct network slice life cycle management. Their position in the radar (see Figure 9) makes it clear that the trend is to evolve towards a fully digital OSS stack, wherein design, data management, assurance and orchestration processes all need to be aligned and integrated across physical, virtual and cloud assets. This journey needs to be accompanied with new levels of automation, extending them further up the stack, into the E2E service and network management domain.

The first stop of the journey is the as-is ring of the radar. Today’s OSS consists of isolated, monolithic systems passing through specialized applications that require complex integration. In addition, automation is quite limited (only present in specific parts of the OSS, and not covering all the management domains) and with many silos (part of the automation is often developed as standalone scripts used by separate domain teams, with each team creating and managing automation using its own environment). However, with the edge computing and 5G SA technologies just around the corner, most of the operators have already integrated a **MANO stack** in their OSS, using SOL005 [25]. Apart from the monetization of Infrastructure as a service (IaaS) services, the MANO stack allows operators to move carrier-grade functions and services to the cloud, leveraging NFV technology. This is critical for a cost-efficient delivery of some NSA slicing solutions, like the ones represented in Figure 10C–E, where there is a need to deploy multiple instances of the same network functions.

The second stop is the deploy ring of the radar. Coinciding with the early rollout of 5G SA networks and the commercialization of the first B2C slices, operators need to integrate slicing logic into their OSS. The short-term focus is on basic slice provisioning. In this regard, the following solutions are needed.

- **NST/NSST**. The Network Slice Template (NST) and Network Slice Subnet Template (NSST) are pre-configured service descriptors that help create a blueprint to ease replicability (i.e., design once, deploy everywhere) of offered network slices and slice subnets.
  For example, the NSST corresponding to a 5GC slice could include the following information: (i) the type of services that the 5GC slice supports, (ii) the 5GC slice topology, and (iii) the 5GC slice placement policy. Based on the fact that a network slice subnet can be deployed as an ETSI NFV network service (see Figure 1), the information given in (ii) is a pointer to the corresponding Network Service Descriptor (NSD), while the information captured in (iii) is the specification of the PoP type where individual network service components can be deployed, and their affinity/anti-affinity rules.
  For the construction of the NST, the approach is similar, but including pointers to the NSSTs.
  The design, development, testing and validation of NSTs/NSSTs/NSDs, and their subsequent onboarding to the catalogs, are activities that formally belong to the network slice preparation phase.
- **Lite Slice NRM fragment**. As seen in Figure 1, the 3GPP information model for network slicing is complex, with a high number of classes and different containment-naming relationships across them. Furthermore, this model is in continuous evolution, as long as the work in 3GPP SA2 and GSMA GST/NEST evolves. For this reason, it is preferable to have a lite version of the slice NRM fragment in the short term. This lite version may contain (i) all classes, except the EP_Transport IOC, which is intended for Rel-16; (ii) simple relationships across them, leveraging as much as possible on 1:1 mappings; and (iii) a limited number of attributes in the ServiceProfile and SliceProfile constructions. In these constructions, only the functionality and performance related attributes that are needed to provision 5GC type A2 slices (B2C market) will be implemented.
  In case of selecting different vendors for the NSMF (in charge of interpreting ServiceProfile attributes) and NSSMF (in charge of interpreting SliceProfile attributes), the operator must ensure the compatibility of the lite slice NRM fragment with these NSMF and NSSMF solutions.
- **Baseline Decision Engine**. The Decision Engine is the OSS component in charge of performing the feasibility check procedure (see Figure 2). In network slicing, this procedure makes use of three input data: (i) the service requirements that the slice must support, captured in the ServiceProfile; (ii) the NST, stored in the catalog; and (iii) the resource and network status, stored in the inventories. As detailed in Section 2.4, the feasibility check is a two-step operation. If the outcome of this operation is ‘feasible’, the decision engine uses internal policies and optimization algorithms to decide on the placement and resource allocation of the requested slice, and send out the decision to the NSMF, which enforces it. The format of this decision is as follows: *instantiate a new slice, configuring the NG-RAN slice with the radio resource allocation solution “X”, and deploying the 5GC slice in this PoP “Y”. For the instantiation of the 5GC slice, use the deployment flavor “Z” from the NSD referred in the NSST*.
  In the short term, it is recommendable to use a baseline Decision Engine. This solution, captured in the deploy ring of the radar, assumes that the logic of the Decision engine is rather simple, built upon simple static rules or simply betting on trial-and-error approaches. The reason is that the variety of 5GC slice types is expected to be rather low, and all targeted for B2C market; therefore, there are no really a higher number of deployment options to choose from.

In the medium term, the upgrade to Rel-16 will allow operators to start commercializing network slicing for the B2B customers, with the 5GC slice types B1, B2 and B3. The shift from B2C to B2B market requires a much more rich-featured OSS than before, with better capabilities in terms of provisioning and scalability, and with the integration of first assurance mechanisms. On the one side, the enhancement in provisioning and scalability is due to the higher variety of slices, with tens of instances running in parallel. On the other side, the introduction of assurance features will allow operators to guarantee the QoS and contracted SLAs of each network slice, much more business-critical than those for B2C slices. The test ring of the radar captures the solutions that are needed to cope with the new OSS needs. These include:

- **Complete Slice NRM fragment**. The new wave of slice offerings will be made available in the operator’s service portfolio, with the publication of different NESTs. The wide variety of NEST parameters that can be configured by the customer prior to issuing service order, makes it necessary to evolve Slice NRM fragment. The main focus will be on the ServiceProfile and SliceProfile constructions, which need to extend their attributes to make them mappable to NEST parameters. Other minor enhancements are also expected, for example the addition of EP_Transport IOC and more flexible class relationships, as depicted in Figure 1.
- **Advanced Decision Engine**. This solution is based on evolving the logic of the Decision Engine, with the integration of multi-objective policies and optimization algorithms. With many more slices running in parallel, each with completely different requirements, the operator’s system becomes much more dynamic. If changes are too quick, the stability of the operator’s network could be compromised. To avoid this, it is critical that the decision on slice placement and resource allocation (i) minimizes the probability of modifying the slice at operation time, and (ii) optimizes resource usage.
- **Baseline slice assurance**. The assurance represents the ability of the operator to retrieve management data from individual slices, using them as input for SLA verification. Examples of these data includes S-NSSAI level information on slice status (e.g., activated, de-activated), performance measurements and KPIs (e.g., UL/DL throughput, latency and packet loss rate) [104,105], notifications on fault events and alarms (e.g., threshold crossing) [106] and trace data.
  The operator may start with S-NSSAI level management data based on the aggregation of metrics collected from the 5GC (via NWDAF) and NFV MANO (via SOL005). These data will then be aggregated to check the health of the slice, verifying whether it meets the SLA requirements. In case of SLA violation, the operator will trigger corrective/remediation actions (e.g., capacity increase, re-configuration) on the corresponding slice. The number of actions available by then is expected to be limited, according to the experience the operators are getting from the trials.
  In this baseline assurance solution, it is assumed that the assurance group in the OSS (see Figure 2) will include the ‘data aggregation’ and ‘Service Quality Management’ modules, but not the ‘AI models and training’ module.

Finally, in the longer run (explore ring in the radar), the operator focus will be on having an **advanced slice assurance** toolkit available in their OSS stack. To that end, operators are scouting solutions in two main directions. On the one hand, the incorporation of the RIC and the SDTN controller as new data sources, building on the two already in place (NWDAF and NFV MANO). To transform these quite different types of data into useful information for the Service Quality Management activities, the data aggregation framework may leverage on the Management Data Analytics Service (MDAS) functionality, reported by 3GPP in [107,108]. On the other hand, the full automation of the assurance pipeline (data collection and aggregation → insights → corrective/remediation actions), using zero-touch mechanisms such as those outlined in [109]. For the insights step, the ‘AI models and training’ module is to be used.

Once all the solutions in the radar have been discussed, one can wonder how the operators can assess the level of maturity of their OSS throughout this journey. This can be done using different OAM related KPIs, including scalability related KPIs (e.g., number of slice types, number of instances running in paralell), commissioning related KPIs (e.g., network slice instantiation time) and operation related KPIs (e.g., network scaling re-configuration time and scaling in/out time), among others.

### 8.2. Capability Exposure

Capability exposure represents the ability of a network slice provider to securely expose capabilities from its managed functions and services towards one or more authorized customers. These capabilities are made available for consumption through easy-to-use service APIs. When addressing customers with limited to no telco experience, it is important that the offered service APIs focus on the customer industry segment and hide the complexity of the underlying network. This requires the provider to abstract and combine low-level network APIs (e.g., 3GPP, ETSI and IETF stage 3 solutions) into customer-facing and intent-based APIs.

**Figure 18:**
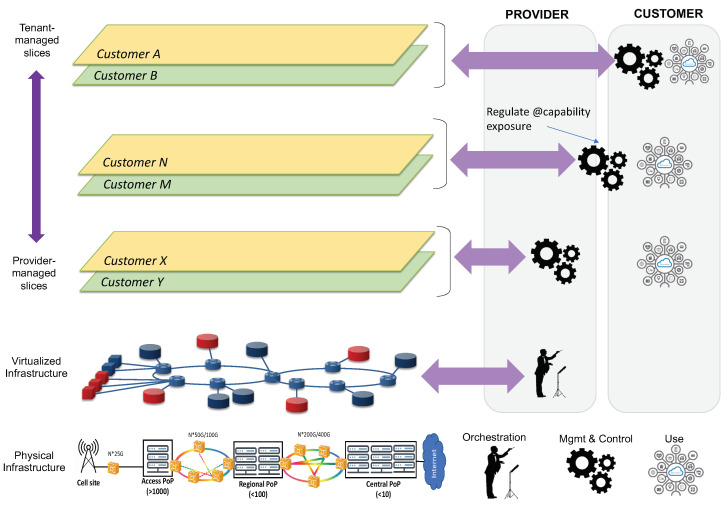
Capability exposure in network slicing environments.

The importance of capability exposure in slicing environments has been already highlighted in [110,111]. When becoming a network slice provider, the operator can leverage this feature to offer NSaaS in multiple forms, from *provider-managed slices* (i.e., the provider is in charge of the slice operation, while the customer can merely use the network resources of the provider slice, without any further capability of managing or controlling it) to *tenant-managed slices* (i.e., the customer takes full control of the slice, and the provider just segregates the infrastructure as required for that purpose) [112], with some variants in between. By regulating the exposure, the operator can define the visibility and the degree of control the customer can take over the slice. Figure 18 shows the logic behind the capability exposure concept.

In the following, we present the service APIs we foresee the operator may offer for capability exposure. For the sake of simplicity, these APIs have been clustered into API families.

Today’s situation is as follows: only some operators have NSA slices, based on any of the technology solutions captured in Figure 10. These slices are rather static, with everything configured beforehand; this means that no further customization is allowed from the customer side. In this context, the only actions that the customer can take are (i) to access the operator’s portfolio, browse the service offering, select the NSA slices and issue a service order; (ii) to get high-level data on NSA slice status, for the purpose of SLA assurance; and (iii) to get information about the usage of the NSA slice and components that can be charged for. All these capabilities are provided with service APIs belonging to the **Accounting, Charging and Billing** API family. As seen, the possibilities with this as-is solution is quite limited, as it only permits provider-managed slices. However, this situation may radically change with the shift towards SA networks.

In the short term (deploy ring in the radar), the focus will be on premium B2C users, as noted in Figure 11. For the provision of Type A2 slices, the operator will bet on internal NESTs. With the presence of NEF in the Rel-15 5GC solution suite, and the integration of the first Rel-15 features in their OSS systems, the operators are starting to launch a new API family: **Device Info**. It provides the customer with the ability to receive information related to one or more devices. This includes information on device subscription (e.g., subscribed NSSAI), device location tracking (UE location and cell site), device mobility (handovers), device status (e.g., serving S-NSSAI, loss of connection, etc.) or CN type change (5GC to EPC coverage, and vice versa). This API family might offer the customer the possibility to explicitly query for this information (request-response mode) or be reported with notifications on subscribed events (subscribe-notify mode).

In the medium term (the test ring in the radar), the operators will publish the NESTs into their service portfolio, once 5GC capabilities necessary for building up B2B category slices are available. The use of NESTs provides an easy solution for the customer to request the allocation of a slice. Based on the service requirements captured in the NEST, the operator can then decide which B2B category slice is most appropriate, and provision it. In this time frame, it is also expected that customers gain experience with slicing technology, and hence want to retain more control of their slice. This requires the operators to increasingly expose further capabilities, with the definition of new API families. Below, there is a list of API families that some tier-1 operators have started to test and validate with some industry verticals and hyperscalers.

- **Edge computing**: these service APIs are available for those customers that want to extend their allocated slices with third party applications, with these applications hosted by the telco edge cloud. In a nutshell, this API family provides three main capabilities: (i) edge node discovery capability, which allows the customer to discover the set of edge nodes available in a certain region; (ii) edge node profile capability, whereby the customer can get information on capabilities and supported features of a given edge node, so as to check whether this node is valid for hosting the application; (iii) application resource allocation capability, that allows the customer to request the operator to deploy the application on a given edge node.
- **Device configuration**: provides the customer with the ability to register a device into the network slice, and to update device subscription. Based on the subscription information received by the customer, the operator updates the Unified Data Management (UDM) accordingly. This API family is quite useful for (industrial) IoT scenarios, where B2B customers want to retain control of the configuration of their devices. For further information on UDM functionality, see Figure 4 and [10].
- **Network slice management data**: with this API family, the customers can subscribe to receive management data related to their allocated slices, at per S-NSSAI level. The individual customers might also choose how they want to consume these data, according to their preferences. For example, regarding performance management data, a customer could specify the KPIs to be informed, the batch format and the reporting period.
- **QoS control**: provides the customer with the ability to set and modify quality for a slice (e.g., maximum latency, guaranteed throughput, maximum admissible packet rate), on demand. The operator captures the customer-triggered request and routes it through the NEF down to the PCF, which will ultimately set/modify the 5QI associated with the in-slice PDU session(s).
- **Traffic influence**: provides the customer with the ability to modify the connection policies of UEs attached to the slice, in terms of how the traffic flows. For example, if the customer has deployed a 3rd party application in a specific edge node, with this API family the customer can then request the re-routing of the in-slice packet flows to this edge node.

Finally, in the longer run, the integration of Rel-17+ features into the network and OSS layers will allow operators to unleash the full potential of NSaaS. Examples of API families that operators foresee to make available in five-to-seven years time are captured below.

- **Slice day-2 configuration**: allows the customer to retain full control of slice day-to-day operation, which is the ultimate realization of tenant-managed slices. With this API family, the customer could request topology changes and resizing (e.g., scaling in/out) on the slice, based on the processing of alarms and KPIs received.
- **Customer-defined slice composition**: allows the customer to request a slice *à la carte*. To enable this feature, the operator shall define a marketplace with different functions and applications. The customer should be able to browse this marketplace, design an E2E slice by connecting selected functions/applications (following a ‘plug-and-play’ approach), and order its provisioning. This is a giant step forward, and requires thinking of novel ways of designing slices and capturing their service requirements, beyond today’s NEST approach.

## 9. Conclusions

As 5G standards get completed and commercial products mature, operators shall demonstrate their ability to efficiently combine available technology solutions to deliver network slices for different customers, with a number of these slices being short-lived and provisioned on demand. Network slicing may allow network operators not only to achieve relevant CAPEX and OPEX reductions in their managed network infrastructure, but also to significantly enrich their portfolio with innovative service offerings, which is a key differentiator in the highly competitive environment the provision of network services has become today.

Network slicing is an E2E solution that has an impact on all subsystems, including the different technology domains (including RAN, CN and TN) and the operational systems (the OSS). The main problem is that the degree of maturity of slicing support is quite different in these subsystems. Indeed, while the CN is the technology domain that currently supports the most advanced slicing capabilities, the TN is still in and early phase, with RAN domain sitting in between. In the case of the OSS, in charge of the management of these three technology domains and of the orchestration activities across them, we can find different paces of technology evolution.

According to the above rationale, it is clear that it may take several years for operators to prepare their systems to fully harness the power of network slicing, and become able to support NSaaS. Awaiting this moment to start commercializing and monetizing network slicing is not an option, either for operators (who cannot offset the costs associated with the introduction of slicing capabilities into their assets) or for customers (which may not need the full set of capabilities for their use cases from the very beginning). In this context, what the industry demands is a phased-based rollout of slicing, starting with early-stage solutions and outlining a vision of what is desired in the longer run to guide progress and focus.

In this article, we have presented a radar with the mission to help industry in defining this rollout. This radar captures a complete landscape of network slicing solutions, linking these solutions to different timelines based on their technical viability and market demands. In addition to this timing, the radar has also outlined the dimensions that have an impact on the usability (how and where) of these solutions, across all operator managed domains. In the RAN domain, network slicing solutions have been discussed based on functional aspects (e.g., disaggregation and O-RAN integration), radio resource allocation and penetration in the operator’s cell footprint. In the CN domain, discussions have been around the fulfilment of isolation and customer requirements of network slice customers, resulting in the use and combination of different 5GC functions, with different profiles. In the TN domain, solutions have been articulated around the technology capabilities available in the underlay WAN, complemented with the automation and programmability capabilities brought by SDN technology. Finally, in the OSS domain, aspects related to network slice OAM and capability exposure have been addressed, with a focus on provisioning and assurance activities.

The network slicing solutions captured in the radar have been analyzed based on their timeline, together with the above dimensions in Section 4, Section 5, Section 6, Section 7 and Section 8. In order to provide the reader with a common reference system to interpret the content of this radar, we started this work by providing the technical background of network slicing (Section 2) and its impact on individual technology domains (Section 3).

We are confident that the radar presented and discussed in this article will be a valuable reference for operators to outline their network slicing rollout plan within their service footprint. In fact, each operator can go through the radar and decide which specific solutions they want to activate in the different domains (CN, RAN, TN and OSS) and how to combine them, so that the resulting E2E capabilities can meet the service requirements of the targeted customers. This work has not entered into these decisions, which are entirely up to each operator, and subjected to certain market and business driven factors. In other words, this article provides the input material that an operator needs for the definition of a network slicing rollout plan (e.g., description, analysis and profiling of network slicing solutions, in terms of timing and features), but with no details on how to execute this plan. Getting to these details would require going through two activities that are out of the scope of this article, and therefore not addressed in the discussion of this work:

- First, the definition of a well-defined methodology for the design of a robust rollout plan. The radar captures all available solutions and categorizes them into different dimensions and timelines; however, it does not provide clues on how an operator shall combine them in production networks. Examples of reference methodologies to this end can be found in [4,5,6]. These technology analyst reports provide high level recommendations and good practices for operators to design their individual rollout plans for network slicing.
- Secondly, the assessment of proposed network slicing solutions, so that their main advantages and disadvantages can be outlined in advance. This needs to be done individually (i.e., per domain-specific solution) and E2E (i.e., based on selecting combinations of RAN+TN+CN solutions). One of the aspects we consider for future work is the evaluation of these solutions, using either simulation work or PoCs, depending on the maturity of each solution.

Besides operators, the outcomes of this work may be of relevance for other audience, including vendors, verticals and researchers. On the one hand, the solutions captured in this radar can help the *vendors* to consolidate the feature roadmap in their products. On the other hand, the *verticals* can use this radar to understand what slicing capabilities may be available for consumption, and by when. This knowledge should be important for them, especially to manage expectations in terms of implementable use cases. Finally, this work provides a reference for *researchers* to keep working on the validation of E2E slicing solutions, using experimental setups that mimic the limitations and conditions of real-world carrier networks.

## Figures and Tables

**Table 1:** Examples of NESTs for the three slices represented in Figure 2.

GST Attribute	Network Domain			mIoT NEST	eMBB NEST	uRLLC NEST
	RAN	TN	CN			
Availability	X	X	X	99.9	99.99	99.9999
Session and Service Continuity (SSC) support			X	SSC Mode 1: the IP address is preserved	SSC Mode 1: the IP address is preserved	SSC Mode 1: the IP address is preserved
Maximum DL (UL) throughput per UE	X		X	2 (4) Mbps	200 (200) Mbps	40 (40) Mbps
DL (UL) throughput per slice	X	X	X	Maximum: 30 (60) Gbps	Guaranteed: 300 (200) Gbps	Maximum: 20 (20) Gbps
Maximum number of PDU sessions			X	500,000	80,000	1500
Slice Quality of Service (QoS)	X	X	X	3GPP 5QI: 9	3GPP 5QI: 1,2,5,6,7,8,9	3GPP 5QI: 82
Maximum supported packet size	X	X	X	300 bytes	1500 bytes	160 bytes
UE density (per km^2^)	X		X	100,000	500	80
Simultaneous use of the slice	X	X	X	Can be used simultaneously with any slices with same SD value but different SST value	Can be used simultaneously with any slices with same SST value but different SD values	Cannot be used simultaneously with any another slice
Supported device velocity	X		X	10 km/h-Pedestrian	500 km/h-High speed vehicular	120 km/h-Vehicular

NOTE 1: Slice QoS parameters attribute defines all the QoS relevant parameters supported by the network slice, including priority level, packet delay budget (i.e., maximum allowed latency), packet error rate, jitter and maximum packet loss rate. These attributes are indexed using a 3GPP defined scalar called 5G Quality Indicator (5QI). For further details on 5QIs, see [10]. NOTE 2: The maximum number of PDU sessions attribute describes the maximum number of concurrent PDU sessions supported by the network slice. To enforce this quota on the slice, the 5GC network function called Network Slice Access Control Function (NSCAF) is required. For further information of this function, see Section 5.2.

**Table 2:** RAN slice characterization based on configured quotas.

Ded Min Slice Quota	Minimum Slice Quota	Maximum Slice Quota	RAN Slice Characterisation
10%	10%	45%	Dedicated slice-profile 1 (dedicated + shared)
10%	20%	45%	Dedicated slice-profile 2 (dedicated + priori-tized + shared)
-	20%	45%	Prioritized slice (prioritized + shared)
-	0%	45%	Best effort slice (can also use prioritized re-sources if spare left by another prioritized slice)

**Table 3:** A comparative analysis of RAN slicing mechanisms.

Topic	Priority Scheduling	Radio Resource Partitioning
Solution	All slices share resources, and the slice SLA is guaranteed by increa- sing the scheduling priority of devices that have not reached the minimum guaranteed rate	Reserve dedicated resources and prioritized resources for specific slice(s) to ensure that devices in the slices have sufficient available at any time
Guarantee	Best effort SLA guarantee: when the cell is congested, the SLA of some slices may not be guaranteed	Customers get accurate SLA guarantee
Isolation	Resource sharing between slices that can only provide limited soft isolation with different priorities	Hard isolation of resources between slices avoids mutual influence across slices and provides an isolation effect similar to that of dedicated frequency
Scenario	B2C market slicing	B2B market slicing

**Table 4:** Overview of network YANG models for the NBI of per-technology domain SDN controllers.

Domain Controller	NBI Models	Description
IP controller	L3SM [91] L2SM [92] L3NM [93] L2NM [94]	These models are used for the provisioning of L2/L3 connectivity services.They describe a VPN service from the customer (LxSM) or network operator (LxNM) viewpoint
	TE [95] TE Topology [96]	These models allow to manipulate Traffic Engineering (TE) tunnels. There exist extensions to work with a desired technology (e.g., MPLS RSVP-TE tunnels, Segment Routing paths).
Optics controller	T-API [97]	It is the model with higher market adoption. It includes technology-specific information from each transport layer, including DSR/Ethernet, OTN/ODU and photonics media.
Microwave controller	OpenBackhaul model set [98]	This repository captures the set of models for the microwave segment. They are extensions from those captured in ONF Core Information Model [99].

## Data Availability

Not applicable.

## Abbreviations

The following abbreviations are used in this manuscript:

5G	Fifth Generation
5GC	5G Core
5QI	5G Quality Indicator
AI	Artificial Intelligence
AMBR	Aggregate Maximum Bit Rate
AMF	Access and Mobility management Function
API	Application Programming Interface
ASIC	Application Specific Integrated Circuit
B2B	Business-to-Business
B2C	Business-to-Customer
BSS	Business Support System
CN	Core Network
CSMF	Communication Service Management Function
CU	Centralized Unit
DN	Data Network
DRB	Dedicated Radio Bearer
DSCP	DiffServ Code Point
DU	Distributed Unit
DWDM	Dense WDM
E2E	end-to-end
eCPRI	Enhanced Common Public Radio Interface
EPC	Evolved Packet Core
Flex-E	Flexible Ethernet
Flex-O	Flexible Optical Transport Network
FPGA	Field Programmable Gate Array
GBR	Guaranteed Bit Rate
GSMA	GSM Alliance
GST	Generic network Slice Template
IaaS	Infrastructure as a Service
IOC	Information Object Class
L4S	Low Latency, Low Loss, Scalable throughput
LSP	Label Switched Path
MANO	Management and Orchestration
MBR	Maximum Bit Rate
MDAS	Management Data Analytics Service
MPLS	Multiple Protocol Label Switching
MW	Microwave
near-RT RIC	near-Real Time RIC
NEF	Network Exposure Function
NEST	Network Slice Type
NFV	Network Functions Virtualization
NFVO	NFV Orchestrator
NG-RAN	Next Generation RAN
non-RT RIC	Non-Real Time RIC
NPN	Non-Public Network
NR	New Radio
NRM	Network Resource Model
NSA	Non Standalone
NSaaS	Network Slice as a Service
NSCAF	Network Slice Access Control Function
NSD	Network Service Descriptor
NSI	Network Slice Instance
NSMF	Network Slice Management Function
NSSAI	Network Slice Selection Assistance Information
NSSI	Network Slice Subnet Instance
NSSMF	Network Slice Subnet Management Function
NSST	Network Slice Subnet Template
NST	Network Slice Template
NWDAF	Network Data Analytics Function
OAM	Operation, Administration and Maintenance
OS	Operation System
OSS	Operations Support System
PCE	Path Computation Element
PCF	Policy Control Function
PDU	Packet Data Unit
PLMN	Public Land Mobile Network
PNI-NPN	Public Network Integrated NPN
PoC	Proof of Concept
PoP	Point of Presence
PRB	Physical Radio Block
RAN	Radio Access Network
RIC	RAN Intelligent Controller
ROADM	Reconfigurable Optical Add-Drop Multiplexer
RRM	Radio Resource Management
RU	Radio Unit
S-NSSAI	Single Network Slice Selection Assistance Information
SA	Standalone
SBA	Service Based Architecture
SBMA	Service Based Management Architecture
SD	Slice Differentiation
SD-WAN	Software Defined WAN
SDN	Software Defined Networking
SDO	Standards Development Organization
SDTN controller	Software Defined Transport Network Controller
SLA	Service Level Agreement
SMF	Session Management Function
SNPN	Standalone NPN
SR	Segment Routing
SST	Slice/Service Type
T-NSC	Transport Network Slice Controller
TAC	Tracking Area Code
TAI	Tracking Area Identifier
TE	Traffic Engineering
TN	Transport Network
TSN	Time Sensitive Network
UE	User Equipment
UPF	User Plane Function
URSP	UE Resource Selection Policy
VLL	Virtual Leased Line
VNF	Virtualized Network Function
VPLS	Virtual Private LAN Service
VPN	Virtual Private Network
VPRN	Virtual Private Routed Network
WAN	Wide Area Network
WDM	Wavelength Division Multiplexing
XR	Immersive Reality
YANG	Yet Another Next Generation
